# Application of bioinformatics analysis and molecular docking to study the mechanism of Qingying decoction in treating psoriasis

**DOI:** 10.1186/s41065-025-00421-8

**Published:** 2025-04-07

**Authors:** Cuicui Shen, Xuewei Liu, Huangchao Jia, Wenhe Wang, Xiaomeng Wang, Haiyan Wang, Dan Wang, Jianwei Li

**Affiliations:** Department of Dermatology, The First Affiliated Hospital of Henan University of Traditional Chinese Medicine, Zhengzhou, 450000 Henan China

**Keywords:** Qingying decoction, Psoriasis, Network Pharmacology, Keratinocyte hyperplasia, Inflammation

## Abstract

**Background:**

Qingying decoction (QYD) is a traditional prescription in China that has been shown to be effective in treating psoriasis. However, its mechanism of action remains to be elucidated.

**Methods:**

The active ingredients and targets of QYD were obtained from TCMSP database, HERB database and SwissTargetPrediction database, respectively. Differential expression gene (DEGs) analysis and weighted gene co-expression network analysis (WGCNA) were used to identify key genes associated with psoriasis. Protein-protein interaction (PPI) network was constructed using STRING platform. Gene Ontology (GO) and Kyoto Encyclopedia of Genes and Genomes (KEGG) analyses were performed using the DAVID database and the clusterProfiler package of R software. Cytoscape 3.9.0 software was used to screen the key components of QYD and the hub targets. Molecular docking was used to detect the binding ability between key components and hub targets. An in vitro model of psoriasis was established by stimulating keratinocyte HaCaT with a mixture of five pro-inflammatory cytokines (IL-17 A, IL-22, IL-1α, oncostatin M, and TNF-α) (M5). Cell viability and cell cycle were measured using cell counting Kit 8 (CCK-8) and flow cytometry, respectively. Real-time quantitative polymerase chain reaction (qRT-PCR) was used to detect mRNA levels of hub genes, high-proliferation marker keratin 6 (KRT6) and inflammatory factors IL-1β, IL-6 and TNF-α. Protein expression levels of PI3K/AKT/FoxO pathway related targets were detected by Western blot.

**Results:**

A total of 139 active ingredients of QYD were screened in this study, with 1033 targets, 59 of which overlapped with psoriasis-related genes. Quercetin, luteolin, kaempferol, beta-sitosterol and methylophiopogonanone A were considered to be the key ingredients of QYD in the treatment of psoriasis. CDC25A, TOP2A, NEK2 and CCNA2 were identified to be the hub targets. QYD could probably regulate cell cycle, T cell receptor signaling pathway and metabolic pathway to treat psoriasis. The key components of QYD had good binding affinity with hub target proteins. QYD significantly attenuated M5-induced hyperproliferation and cell cycle progression of HaCaT cells. M5 stimulation significantly upregulates the mRNA levels of CDC25A, TOP2A, NEK2, CCNA2, IL-1β, IL-6 and TNF-α, while QYD treatment reversed this effect. In addition, QYD treatment inhibited the phosphorylation of PI3K and AKT in M5-stimulated HaCaT cells and upregulated p-FOXO1 protein expression level.

**Conclusion:**

QYD can inhibit the excessive proliferation and inflammatory response of keratinocytes by regulating the PI3K/AKT/FoxO pathway, suggesting that QYD may be an attractive prescription for psoriasis.

**Supplementary Information:**

The online version contains supplementary material available at 10.1186/s41065-025-00421-8.

## Introduction

Psoriasis is a common chronic inflammatory skin disease, characterized by a long course of disease and a high recurrence rate, which is difficult to be completely cured [1]. According to statistics, the global incidence of psoriasis is about 2–3% of the world’s population, reaching 8–11% in some Nordic countries [2]. The average age of onset of psoriasis is 33 years old, and the incidence of psoriasis is higher in adults than in children [3]. The pathogenesis of psoriasis is complex, involving the interaction of genetic, immune and environmental factors [4]. The main histological features of psoriasis include abnormal cell proliferation, angiogenesis, hyperkeratosis, hypokeratosis, and inflammatory cell infiltration [5]. Currently, a variety of therapeutic approaches, including topical drugs, phototherapy, systemic therapy, and/or biotherapy, are used in the clinical treatment of psoriasis [6]. However, these approaches do not fully meet the needs of patients, mainly because the various treatments are often accompanied by side effects and a large proportion of patients develop treatment resistance [7]. Therefore, there is an urgent need to develop new, effective treatments for psoriasis with few side effects.

Qingying Decoction (QYD) is a traditional Chinese medicine (TCM) prescription, which consists of 9 kinds of Chinese medicine, such as buffalo horn, raw rehmannia, scrophulariae, bamboo leaf heart, Ophiopogon japonicus, salvia miltiorrhiza, Coptis, honeysuckle and forsythia. QYD has a variety of pharmacological effects, including anti-microbial, anti-inflammatory and antioxidant effects [8, 9]. It has been reported that QYD can effectively ameliorate skin lesions in some patients with psoriasis [8]. However, its mechanism of action in the treatment of psoriasis remains unclear.

Network pharmacology is a powerful method that combines systems biology, pharmacology, computer science and other disciplines to clarify disease targets and molecular mechanisms of drug action [10, 11]. Weighted gene co-expression network analysis (WGCNA) is a bioinformatics tool used to analyze the correlation between genes and clinical features [12]. Molecular docking can predict the binding mode of receptor-ligand complex [13]. The aim of this study was to explore the potential target and mechanism of action of QYD in psoriasis by network pharmacology, WGCNA and molecular docking analysis, and to preliminarily verify it through in vitro assays.

## Materials and methods

### Screening of active ingredients and targets of QYD

According to the screening criteria [oral bioavailability (OB) ≥ 30% and drug-likeness (DL) ≥ 0.18], Traditional Chinese Medicine System Pharmacology Database and Analysis Platform (TCMSP; https://www.tcmsp-e.com/) was searched to screen the potential active ingredients of scrophulariae, salvia miltiorrhiza, Coptis coptidis, honeysuckle and forsythia. In addition, the components of buffalo horn, raw rehmannia, bamboo leaf heart and Ophiopogon japonicus were obtained from HERB database (http://herb.ac.cn/). PubChem database (https://pubchem.ncbi.nlm.nih.gov/) was searched to obtain the SMILES files. SwissTargetPrediction database (http://www.swisstargetprediction.ch/) was used to predict the potential role of targets. The probability > 0 was used as the filtering condition.

### Identification of differentially expressed genes (DEGs) in psoriasis

From Gene Expression Omnibus (GEO) database (https://www.ncbi.nlm.nih.gov/geo/), gene expression profile dataset GSE182740 associated with psoriasis was downloaded. The differentially expressed genes (DEGs) were then identified by GEO2R tool. The threshold values were *P* < 0.05 and|log2 fold change|>1. A volcano map was then constructed using multiple change values and p-values.

### Construction of co-expression network

The WGCNA package (https://cran.rproject.org/web/packages/WGCNA/index.html) was used to construct the gene co-expression networks. To ensure that the connections between genes match the scale-free network distribution, the WGCNA algorithm selected the results that best fit the scale-free network distribution by choosing the weighting parameters. The soft threshold power β selected by the “pickSoftThreshold” function was used to achieve scale-free topology. In this study, the topological overlap matrix was reconstructed by computing topological overlap measure (TOM), which was a robust measure of network interconnectedness. According to the dissimilarity matrix, which represented the connection relationship of genes, cluster analysis of genes by dissimilarity was performed to construct a hierarchical cluster tree [14]. The dynamic tree-cut algorithm method was adopted to identify the module of gene co-expression with values maxBlockSize = 6000, minModuleSize = 30 and mergeCutHeight = 0.2. Module eigengene (ME) referred to the first principal component of each gene module, and the expression of ME was considered to represent all genes in a module. The most important module could be identified by calculating the correlation coefficient between ME and the trait of interest. In the present study, the trait was defined as inflammation status. With HALLMARK_INFLAMMATORY_RESPONSE (200 genes) from Gene Set Enrichment Analysis (GSEA) database (https://www.gsea-msigdb.org), the inflammation score of each sample was calculated with single-sample gene set enrichment analysis (ssGSEA) [15].

### Screening of candidate targets for QYD treatment of psoriasis

The DEGs, which were regarded as psoriasis-related genes, and the targets of the active ingredients of QYD were imported into the Draw Venn Diagram (http://bioinformatics.psb.ugent.be/webtools/Venn/) online platform for cross analysis, in order to obtain QYD’s potential targets for psoriasis treatment.

### Immunoinfiltration analysis

Immune cell infiltration in each group were calculated using the CIBERSORT algorithm and single-sample gene set enrichment analysis (GSEA) (ssGSEA), respectively [15, 16].

### Construction and analysis of “TCM-component-intersection target” network

Cytoscape 3.9.0 software was used to construct the “TCM-Component-intersection target” network diagram, and the degree value of network nodes was calculated by CytoNCA plug-in, so as to screen out the key components of QYD in the treatment of psoriasis.

### Construction and analysis of protein-protein interaction (PPI) network

QYD’s potential targets in psoriasis treatment were imported into STRING database (https://cn.string-db.org/) to construct the PPI network. The species were set to “homo sapiens”, and the required minimum interaction score was set to “medium confidence (0.4)”. After removing the free points, the PPI network was exported and the TSV file was downloaded and saved. Molecular Complex Detection (MCODE) plug-in was used to filter the important modules of the PPI network. Parameters were set as follows: degree cutoff = 2, node score cutoff = 0.2, K-score = 2, and max depth = 100.

### Enrichment analysis of cross targets

Database for Annotation, The Visualization and Integrated Discovery (DAVID) database (https://david.ncifcrf.gov/tools.jsp) was applied for gene ontology (GO) analysis and Kyoto Encyclopedia of Genes and Genomes (KEGG) enrichment analysis. The species was set to “homo sapiens”, with *P* < 0.05 as the screening condition.

### Identification and analysis of hub targets

cytoHubba plug-in in Cytoscape 3.9.0 software was used to applied 9 algorithms including betweenness, closeness, degree, edge percolated component (EPC) and maximal clique centrality (MCC), density of maximum neighborhood component (DMNC), maximum neighborhood component (MNC), radiality, and stress, to evaluate the PPI network. The TOP15 targets obtained by each algorithm were extracted, and then the hub targets were screened using R software UpSet package. GeneMANIA (http://genemania.org/) was used to construct the gene co-expression network of the hub targets. In addition, the R software package clusterProfiler was used to analyze the GO and KEGG pathways of hub genes and their co-expressed genes.

### Molecular docking

Molecular docking was performed with AutoDock Vina v.1.1.2 [17]. X-ray crystal structures of the hub target proteins were obtained from the Protein Data Bank (PDB) database (https://www.rcsb.org/). The water molecules and heteroatoms in the crystal structure of the protein were removed with PyMol software v.2.4.0, and hydrogen atoms were added, and the charge was calculated. In PubChem (https://pubchem.ncbi.nlm.nih.gov/), the key components of the three-dimensional chemical structure was downloaded, and saved as SDF format, and then converted into mol2 format with OpenBabel software version (3.1.1). After converting all protein receptor and molecular ligand files into PDBQT format using AutoDockTools v.1.5.7 software, molecular docking analysis was performed by AutoDock Vina (version 1.1.2) to calculate binding energy. When the binding energy value is < 0, it is considered that protein and molecule can spontaneously bind and interact [18]. Finally, the docking results were visualized in 3D by PyMol software.

### Preparation of QYD water extract

Buffalo horn 30 g, raw Rehmannia 15 g, Genshen 9 g, bamboo leaf heart 3 g, ophiopogon 9 g, salvia miltiorrhiza 6 g, Coptis rhizome 5 g, honeysuckle 9 g, forsythia 6 g were obtained from Department of Pharmacy, the First Affiliated Hospital of Henan University of Chinese Medicine. As previously reported [19], these medicinal materials were mixed, soaked in 2 L water at room temperature, and decocted twice for 1.5 h each time. After decoction, the mixtures were centrifuged (10000 rpm, 30 min), and the supernatant was collected. The supernatant were mixed and evaporated to obtain the powder. The dry powder was dissolved in 200 mg/mL dimethyl sulfoxide (DMSO; Beyotime, Shanghai, China), and filtered by a filter with a pore size of 0.22 μm, and stored at -20℃.

### Cell culture and treatment

Human keratinocyte cell line (HaCaT) was bought from CoBioer (Nanjing, China). The cells were cultured in Dulbecco’s Modified Eagle’s Medium (DMEM; Invitrogen, Carlsbad, CA, USA), placed in a humidified incubator at 37℃ containing 5% CO_2_. As previously reported [20], a mixture of five pro-inflammatory cytokines (IL-17 A, IL-22, IL-1α, oncostatin M, and TNF-α; M5, final concentration of 2.5 ng/ml) was added into the medium to stimulate HaCaT cells, to establish an in vitro psoriasis model.

### Cell viability assay

Cell counting kit 8 (CCK-8; Beyotime, Shanghai, China) was applied to detect cell viability. HaCaT cells were inoculated into 96-well plates at a density of 5 × 10^3^ cells/well and cultured at 37℃ for 24 h. After the cells were stimulated with M5 (2.5 ng/ml), the cells were treated with different concentrations of QYD (0, 100, 200, 300, 400, 500, and 600 µg/mL) for 24 h. Next, 10 µL of CCK-8 solution was added into each well and the cells were incubated at 37℃ for 2 h. Finally, the optical density (OD) was measured at 450 nm with a microplate reader (Dynatech Labs, Chantilly, VA, USA).

### Cell cycle assay

Cell cycle analysis was performed by staining cell DNA with propidium iodide. HaCaT cells were stimulated with M5 (2.5 ng/ml), and then treated with different concentrations of QYD (0 and 400 µg/mL) or or equal volume of solvent for 24 h. The cells were collected, fixed with 70% ethanol and stored overnight at 4 °C. The cells were then washed twice with phosphate buffer saline (PBS). Next, the cells were incubated with propidium iodide solution (50 µg/ml propidium iodide, 5 µg/ml RNase I, 137mM NaCl, 2.7mM KCl, 8.1mM Na_2_HPO_4_ and 1.47mM KH_2_PO_4_) at 37 °C for 30 min. After the cells were washed by PBS, DNA content was analyzed using a FACSCalibur flow cytometer (BD Biosciences, San Jose, CA, USA). Modifit version 3.3 (Verity software House, Topsham, ME, USA) was used to analyze the results.

### RNA extraction and quantitative real-time PCR (qRT-PCR) analysis

HaCaT cells in different groups were collected, and total RNA was extracted and purified with a TRIzol kit (Invitrogen, Carlsbad, CA, USA) according to the manufacturer’s instructions. Total RNA was reverse-transcribed into complementary DNA (cDNA) using a M-MLV reverse transcription kit (Promega, Shanghai, China). qRT-PCR was performed with a SYBR Premix Ex Taq kit (TaKaRa, Dalian, China) on Applied Biosystems System 7500 (Thermo Fisher Scientific, Waltham, MA, USA). Glyceraldehyde 3-phosphate dehydrogenase (GAPDH) was used as internal parameter to calculate the relative expression of target genes by 2^−ΔΔCt^ method. The primers used in this study are as follows: cell division cycle 25 A (CDC25A) forward primer, 5’-GTGGGAGAACAGCGAAGACA-3’, reverse primer, 5’-CAAATAGCGCCTTCACGACG-3’; DNA topoisomerase II alpha (TOP2A) forward primer, 5’-GGGGTCCTGCCTGTTTAGTC-3’, reverse primer, 5’-AGGCTGCAATGGTGACACTT-3’; NIMA related kinase 2 (NEK2) forward primer, 5’-GTTACAGGAGCGAGCGAG-3’, reverse primer, 5’-CTTCAGGTCCTTGCACTTGG-3’; cyclin A2 (CCNA2) forward primer, 5’-GCACTGGTGGTCTGTGTTCT-3’, reverse primer, 5’-GCCAGTCTTACTCATAGCTGACA-3’; keratin 6 (KRT6), 5 ‘-GGGTTTCAGTGCCAACTCAG-3’ (forward) and 5 ‘-CCAGGCCATACAGACTGCGG-3’ (reverse); interleukin-1β (IL-1β) forward primer, 5’-AACCTCTTCGAGGCACAAGG-3’, reverse primer, 5’-AGATTCGTAGCTGGATGCCG-3’; interleukin-6 (IL-6) forward primer, 5’-CCACCGGGAACGAAAGAGAA-3’, reverse primer, 5’-TCTCCTGGGGGTATTGTGGA-3’; tumor necrosis factor-α (TNF-α) forward primer, 5’-GACAAGCCTGTGTAGCCCATGT-3’, reverse primer, 5’-GGAGGTTGACCTTGGTCTGG-3’; GAPDH forward primer, 5’-CACTAGGCGCTCACTGTTCT-3’, reverse primer, 5’-TTCCCGTTCTCAGCCTTGAC-3’.

### Western blot

HaCaT cells were seeded on 6-well plates and cultured at 37℃ for 24 h. The cells were stimulated with M5 (2.5 ng/ml) for 24 h, and then treated with QYD extract (0 and 400 µg/mL) or equal volume of solvent. The cells were lysed on ice with RIPA lysis buffer containing 1 mM phenylmethylsulfonyl fluoride (PMSF) (Beyotime, Shanghai, China) for 20 min and centrifugated at 14,000 rpm and 4 ℃ for 15 min. A BCA kit (Beyotime, Shanghai, China) was then used to detect the protein concentration. Next, for each group, 30 µg protein was electrophoreted in sodium dodecyl sulphate-polyacrylamide gel for 2 h and then transferred to a polyvinylidene fluoride (PVDF) membrane (Millipore, Bedford, MA, USA). The PVDF membrane was blocked with 5% skim milk at room temperature for 2 h. Next, the membrane was incubated with the primary antibody at 4℃ overnight. The primary antibodies included anti-protein kinase B (AKT) antibody (ab8805, 1:1000), anti-phosphatidylinositol-3-kinase (PI3K) p85 alpha (α) antibody (ab191606, 1:1000), anti-phospho (p)-AKT (T308) antibody (ab38449, 1:1000), anti-p-PI3K p85α (Y607) antibody (ab182651, 1:1000), anti-forkhead box protein 1 (FOXO1) antibody (ab39670, 1:1000), anti-p-FOXO1 antibody (ab131339, 1:1000) and anti-GAPDH antibody (ab9485, 1:1000). After three washing with Tween-20 (TBST), the membrane was incubated with secondary antibody (ab205718, 1:5000) at room temperature for 1 h. All of the primary antibodies were from Abcam (Cambridge, UK). The protein bands were detected with a BeyoECL Plus kit (Beyotime, Shanghai, China). ImageLab software version 4.1 (Bio-Rad Laboratories, Hercules, CA, USA) was used for gray density value analysis. GAPDH was used as the endogenous control.

### Statistical analysis

All experiments were conducted independently in triplicate. SPSS 21.0 software (IBM Corp., Armonk, NY, USA) was used for statistical analysis of the data. Data are expressed as mean ± standard deviation (SD). Multivariate comparisons were made using one-way analysis of variance (ANOVA) and Tukey post hoc tests. *P* < 0.05 was considered statistically significant.

## Results

### Screening and target prediction of QYD’s bioactive ingredients

42 ingredients of salvia miltiorrhiza, 12 ingredients of Coptis rhizome, 15 ingredients of honeysuckle and 15 ingredients of forsythia were screened from TCMSP database. In addition, 5 ingredients of buffalo horn, 3 ingredients from Scrophulariae, 30 ingredients from rehmannia, and 31 ingredients from Ophiopogon japonicus were obtained from HERB database. The ingredient of bamboo leaf heart were not extracted. After combining these ingredients and removing the duplicates, 139 potential functional ingredients of QYD were obtained. Basic information about these ingredients is shown in Supplementary Table 1. SwissTargetPrediction database was then used to predict the potential targets of these active ingredients. After screening, merging and removing duplicate values, 1033 drug targets were obtained.

### Identification of key DEGs in psoriasis

**Fig. 1:**
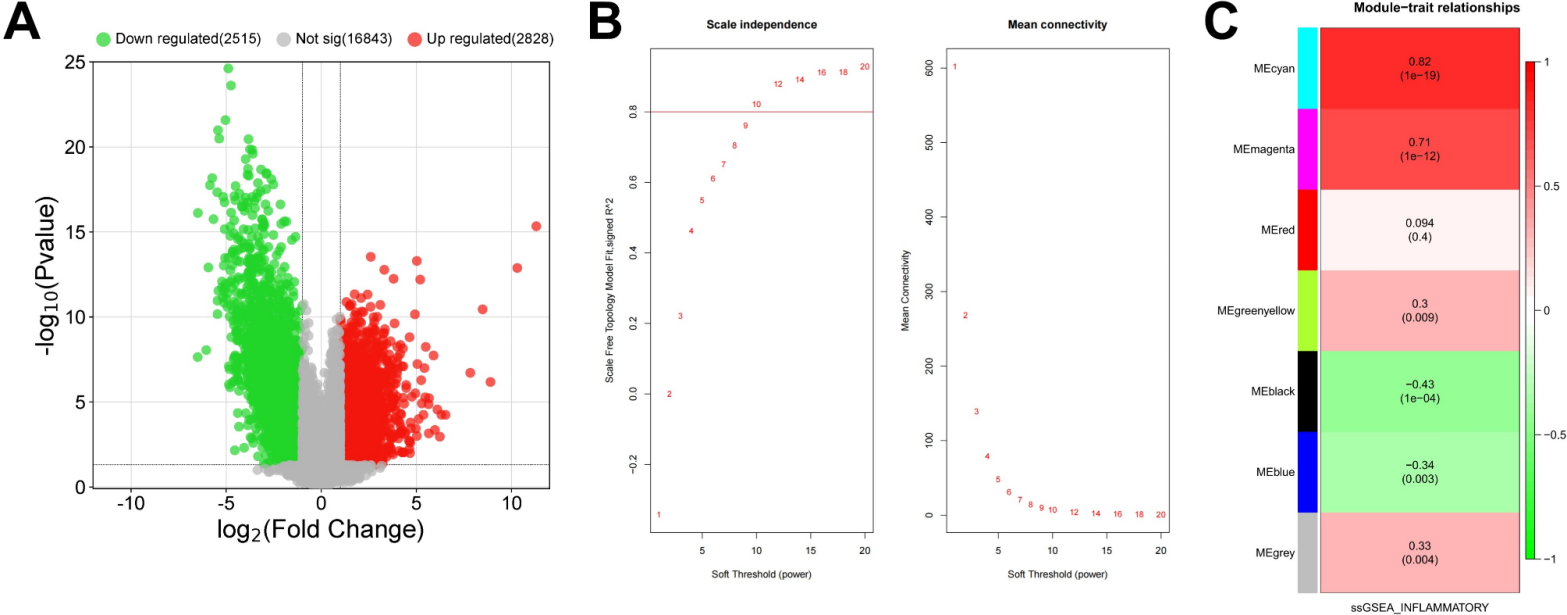
WGCNA was applied to obtain psoriasis/inflammation-related genes. **A**. DEGs of GSE182740 (control group vs. psoriasis group) were screened with *P* < 0.05,|log2 fold change|>1 as the threshold. The horizontal axis is the multiple change in gene expression, and the vertical axis is the statistical significance (p-value). The red dots represent upregulated genes. Green dots represent down-regulated genes. **B**. Soft-thresholding power analysis. R2 > 0.9. **C**. The heat map shows the correlations between the gene module and the trait (inflammation)

To explore the pathogenesis mechanism of psoriasis, gene expression profile data of GSE182740 were analyzed, to obtain the DEGs between healthy controls and psoriasis tissues. The results showed that 2813 genes were up-regulated and 2506 genes were down-regulated in the psoriasis group compared to the control group, suggesting the pathogenesis of psoriasis was driven by multiple genes (Fig. 1A). To identify genes that are significantly associated with psoriasis, we performed WGCNA on these DEGs. A scale-free topological network was constructed with a soft threshold of β = 13 and R^2^ > 0.9 (Fig. 1B). By analyzing the correlation between modules and clinical features, 7 co-expression modules were obtained in the GSE182740 dataset, among which cyan module (*r* = 0.82, *P* < 0.05) and magenta module (*r* = 0.71, *P* < 0.05) showed the strongest positive correlation with inflammation. They contained 47 and 553 genes, respectively (Fig. 1C). We included the genes contained in these two modules in this study, and finally the 600 genes were considered to be psoriasis-related genes.

### Screening the crucial components of QYD in psoriasis treatment

**Fig. 2:**
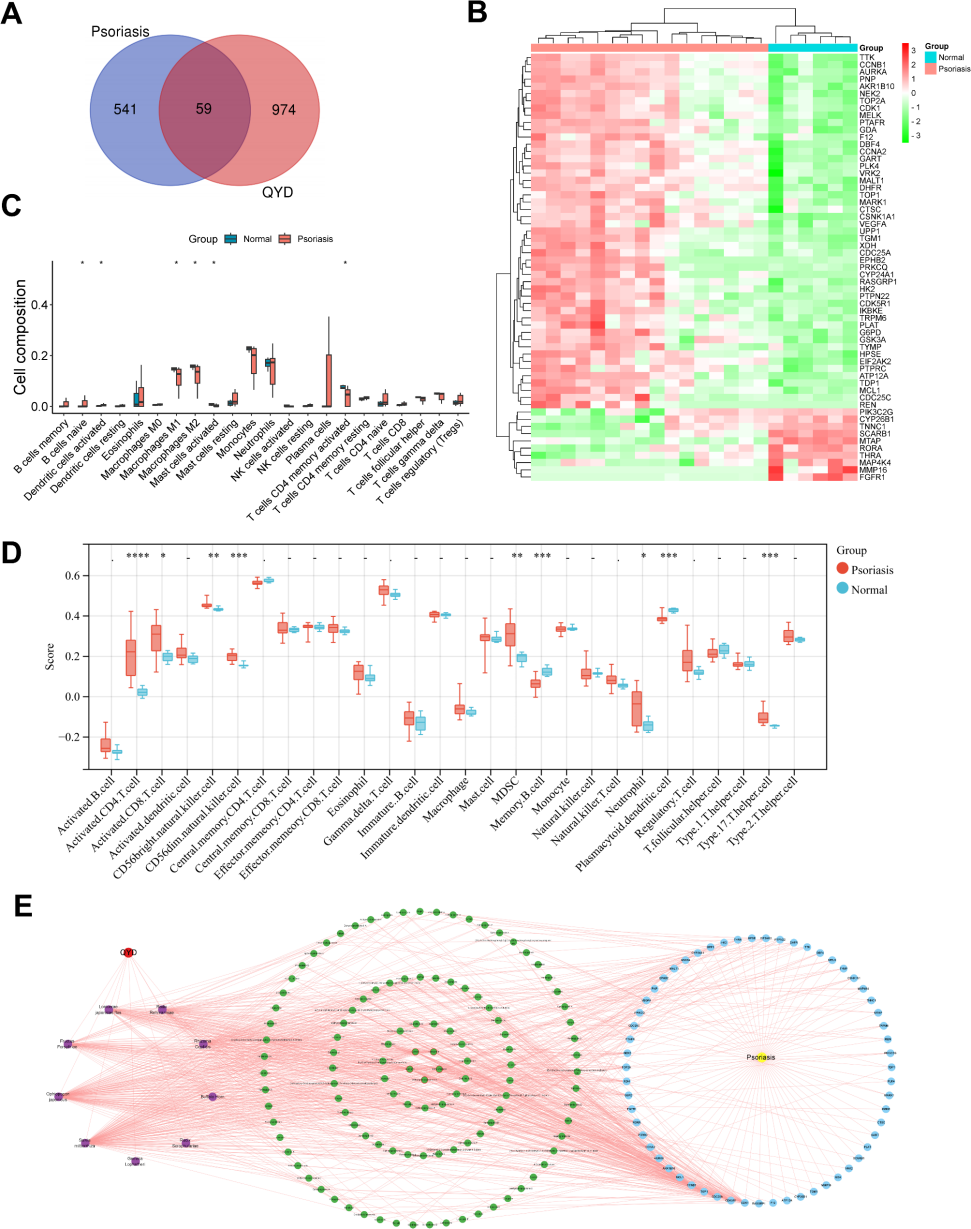
Screening and analysis of QYD targets in psoriasis treatment. **A**. The Venn diagram of the targets of QYD components, and psoriasis-related genes obtained from GSE182740. **B**. The heat map shows the expression pattern of the potential candidate targets of QYD in psoriasis treatment. **C**&**D**. The CIBERSORT algorithm (**C**) and ssGSEA algorithm (**D**) were used to evaluate the difference between control group and psoriasis group, in the infiltration of different immune cell subsets. **E**. The “TCM-component-intersection target” network was constructed using Cytoscape 3.9.0 software. Red circular nodes represent QYD, purple circular nodes represent Chinese medicine, green circular nodes represent ingredients, blue circular nodes represent targets, and yellow circular nodes represent psoriasis. *P < 0.05, **P < 0.01, ***P < 0.001, ****P < 0.0001

To obtain potential targets of QYD in psoriasis treatment, we cross-analyzed 1033 drug targets with 600 psoriasis-related genes. The results showed that 59 targets were identified as candidate target genes for QYD in psoriasis treatment (Fig. 2A). Subsequently, the expression of these 59 genes in psoriasis was analyzed. Compared with healthy controls, 49 genes were up-regulated and 10 genes were down-regulated in the psoriasis group (Fig. 2B). CIBERSORT analysis showed that the infiltration levels of B cells naive and dendritic cells activated in psoriasis group were significantly higher than those in healthy control group. The infiltrating abundance of macrophages M1, macrophages M2, mast cells resting and T cells CD4 memory activated in control group was significantly higher than that in psoriasis group (Fig. 2C, *P* < 0.05). ssGSEA showed that a series of immune cells were dysregulated between the two groups, including activated CD4 T cell, activated CD8 T cell, CD56 bright natural killer cell, CD56 dim natural killer cell, myeloid-derived suppressor cells (MDSC), memory B cell, neutrophil, plasmacytoid dentritic cell and type 17 T helper cell (Fig. 2D, *P* < 0.05). The difference in immune cell infiltration emphasized the importance of immune dysregulation and inflammatory response during psoriasis pathogenesis. In addition, a “TCM-ingredient-intersection target” network was constructed using Cytoscape 3.9.0 software, to identify the crucial functional components of QYD in treating psoriasis. The network contained 185 nodes and 1056 edges (Fig. 2E). The degree value of each node in the network was calculated by CytoNCA plug-in. Quercetin (degree value = 48), luteolin (degree value = 42), kaempferol (degree value = 28), and β-Sitosterol (degree value = 24) and methylophiopogonanone A (degree value = 22), which had the highest degree values, were considered to be the key bioactive ingredients of QYD in psoriasis treatment.

### Construction and analysis of PPI network of candidate targets

**Fig. 3:**
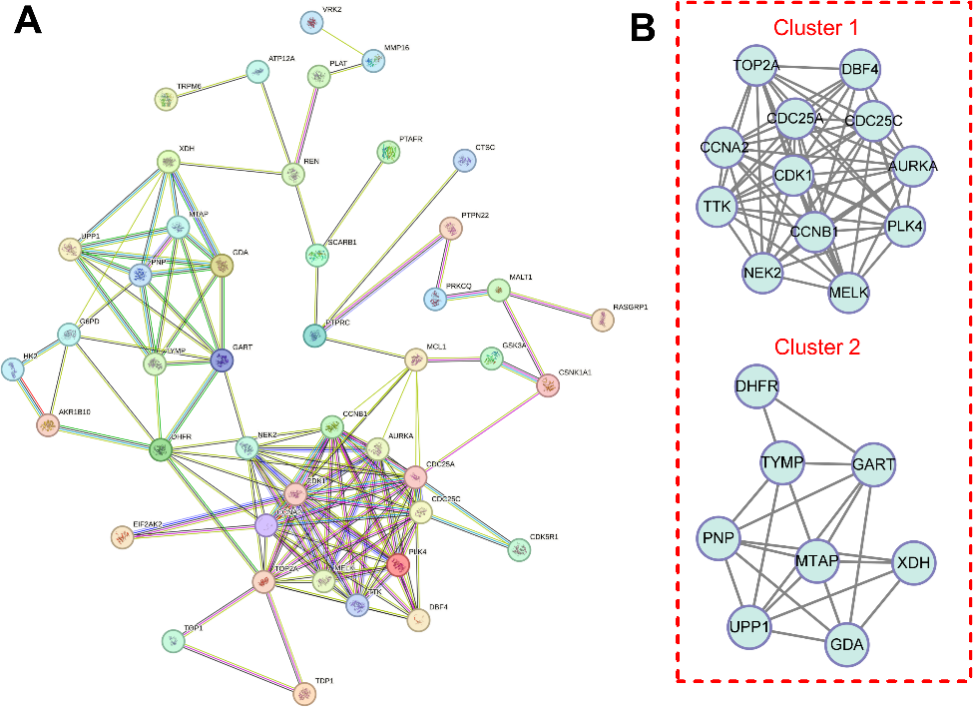
PPI network construction and analysis of potential candidate targets of QYD in psoriasis treatment. **A**. PPI network of potential candidate targets for the treatment of psoriasis. Nodes represent proteins and edges represent protein-protein interactions. **B**. MCODE plug-in was applied to filter highly interconnected clusters in PPI network

In order to investigate the potential mechanism of action of QYD in the treatment of psoriasis, the 59 targets mentioned above were imported into STRING database to construct PPI network. The network consisted of 59 nodes and 130 edges (Fig. 3A). Further, MCODE plug-in was used to perform cluster analysis on PPI network, and two gene clusters for QYD treatment of psoriasis were obtained (Fig. 3B; Table 1). Cluster 1 had higher score, which contains 12 nodes and 63 edges. Interestingly, cluster 1 mainly contained the genes related with cell cycle progression, and cluster 2 mainly contained the genes related with metabolism. This result suggested that QYD probably exerted its therapeutic effect via both repressing abnormal cell proliferation and balancing metabolic homeostasis.

**Table 1:** Clusters of target genes of Qingying Decoction against psoriasis

Cluster	Score (Density*#Nodes)	Nodes	Edges	Node IDs
1	11.455	12	63	DBF4, CDC25A, CDC25C, AURKA, CDK1, PLK4, NEK2, CCNA2, CCNB1, TTK, MELK, TOP2A
2	6	8	21	UPP1, MTAP, PNP, DHFR, GDA, GART, XDH, TYMP

### GO and KEGG enrichment analyses of candidate targets for QYD treatment of psoriasis

**Fig. 4:**
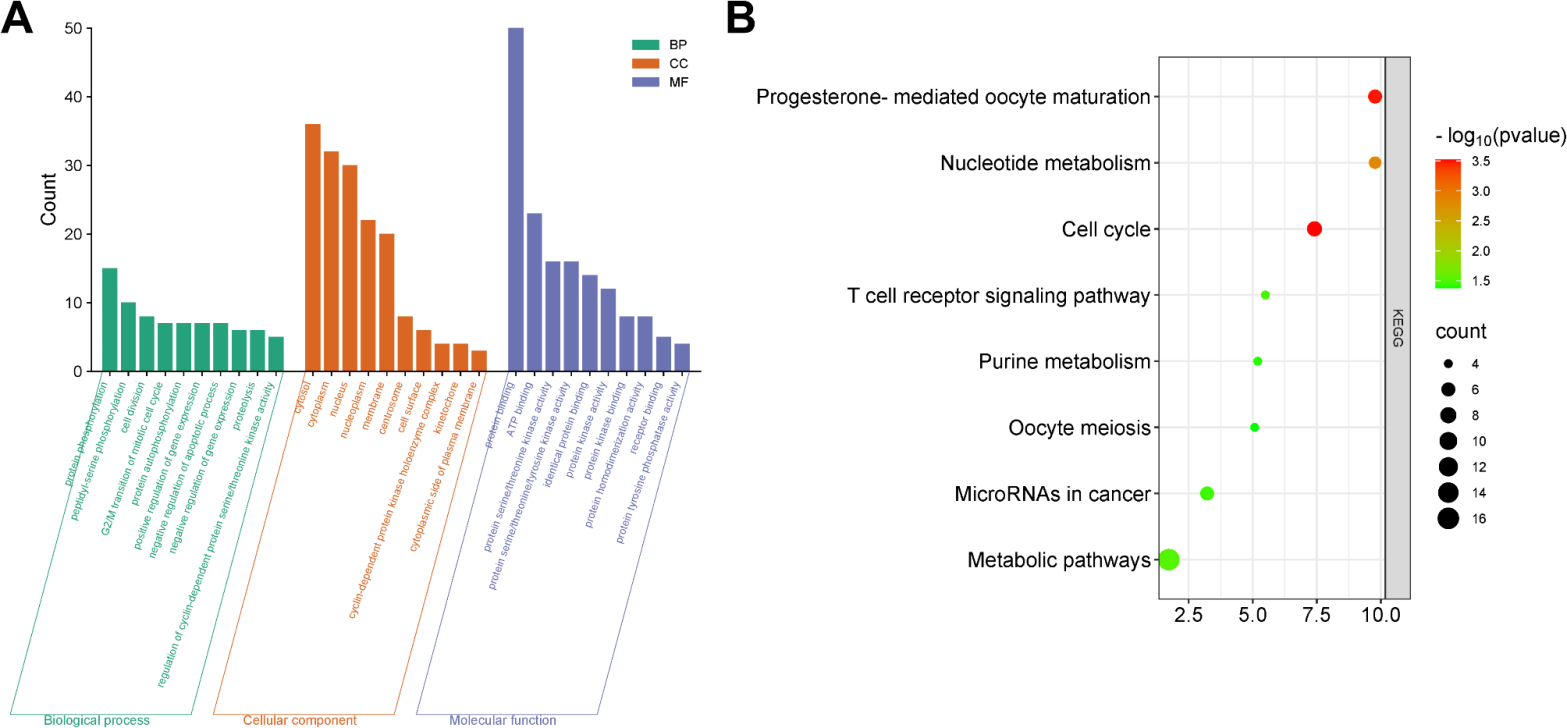
GO and KEGG enrichment analyses of potential candidate targets of QYD in psoriasis treatment. **A**. The histograms show the top 10 biological processes (dark green), cell components (orange), and molecular functions (blue) with the most gene counts of QYD’ targets (*P* < 0.05). **B**. The bubble map is applied to show the results of KEGG enrichment analysis based on QYD’s potential candidate targets in psoriasis treatment. The bubble size represents the gene count, and the bubble color represents the P-value

To explore biological functions of QYD in psoriasis treatment, next, GO and KEGG enrichment analyses of the 59 targets were performed. A total of 103 GO enrichment terms were obtained (*P* < 0.05), including 70 biological processes (BP), 14 cell components (CC) and 19 molecular functions (MF). The top 10 BP, CC, and MF terms with the most gene counts were selected for visualization. As is shown (Fig. 4A), the top 5 terms of BP were protein phosphorylation, peptidyl-serine phosphorylation, cell division, and G2/M transition of mitotic cell cycle and protein autophosphorylation; top 5 CC terms were cytosol, cytoplasm, nucleus, nucleoplasm and membrane; top 5 MF terms were protein binding, ATP binding, protein serine/threonine kinase activity, protein serine/threonine/tyrosine kinase activity and identical protein binding. Through KEGG enrichment analysis, it was found that 8 pathways/biological processes were related to the mechanism of action of QYD in the treatment of psoriasis (*P* < 0.05), They were cell cycle (hsa04110), progesterone mediated oocyte maturation (hsa04914), nucleotide metabolism (hsa01232), metabolic pathways (hsa01100), T cell receptor signaling pathway (hsa04660), microRNAs in cancer (hsa05206), purine metabolism (hsa00230) and oocyte meiosis (hsa04114) (Fig. 4B). These data further supported that abnormal cell proliferation, signal transduction and inflammation were crucial in psoriasis pathogenesis.

### Screening hub targets of QYD in psoriasis treatment

**Fig. 5:**
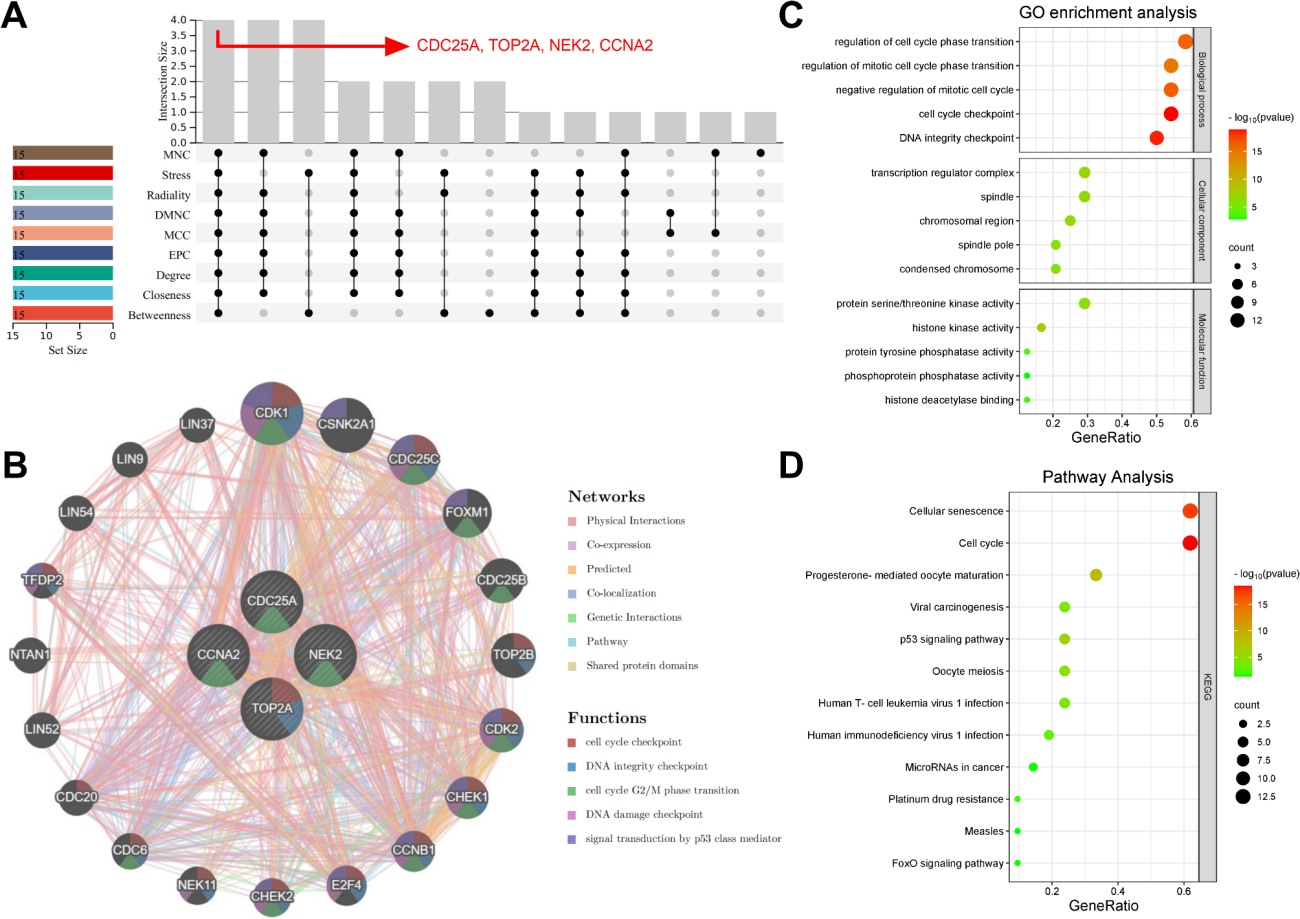
Identification and analysis of hub genes. **A**. Based on the R package “UpSet”, nine algorithms (betweenness, closeness, degree, EPC, MCC, DMNC, MNC, radiality and stress) were used to identify the hub targets of QYD in the treatment of psoriasis. **B**. The co-expression network of hub gene was established by GeneMANIA. **C**&**D**. Based on the co-expressed genes of the hub targets, two bubble maps were used to show the results of GO analysis (C) and KEGG enrichment analysis (D). The horizontal axis is the enrichment score, the vertical axis is the term name, the bubble color represents the P-value size, and the bubble size represents the gene count

**Fig. 6:**
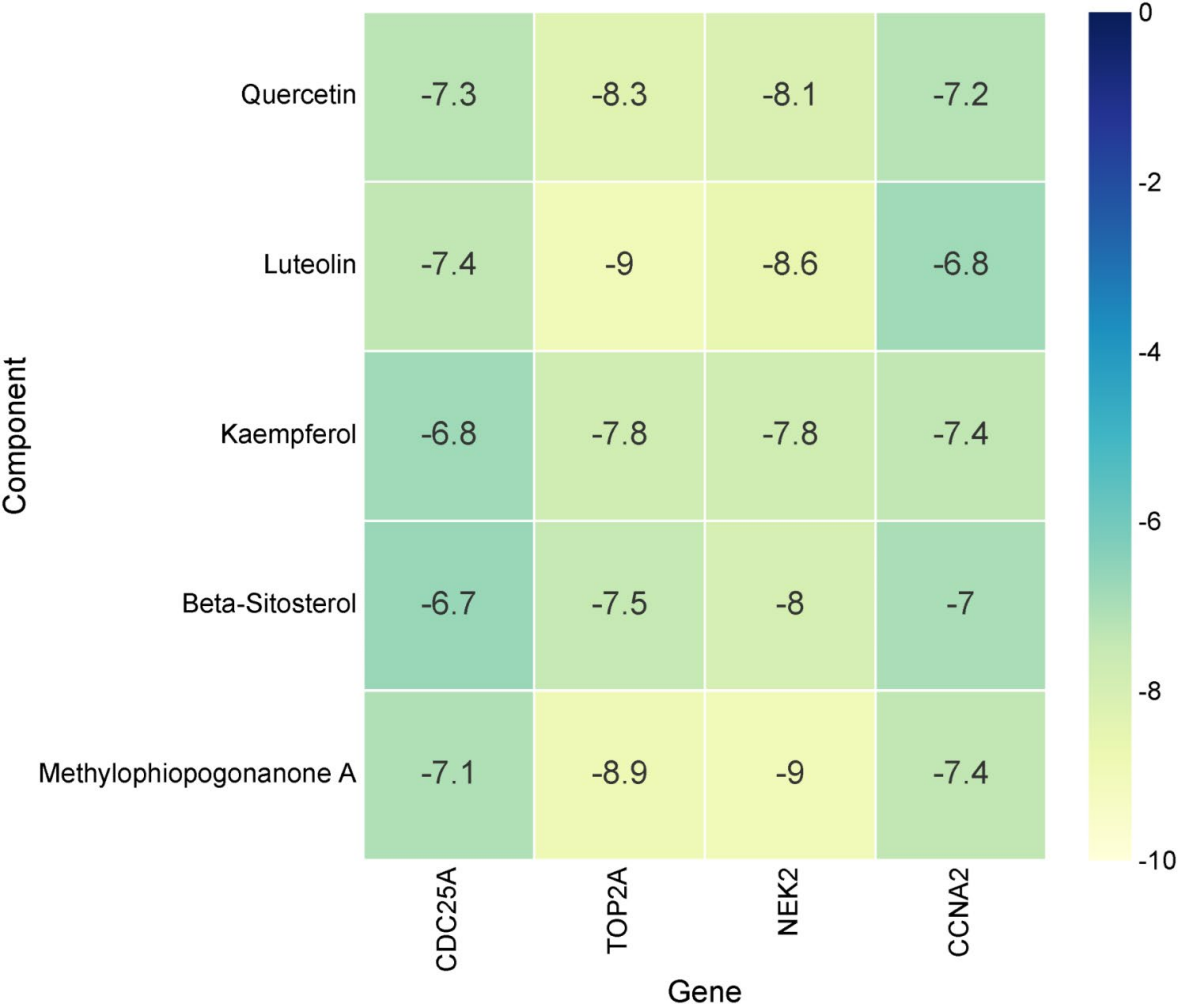
The binding energies between key components quercetin, luteolin, kaempferol, beta-Sitosterol, methylophiopogonanone A with CDC25A, TOP2A, NEK2, and CCNA2

**Fig. 7:**
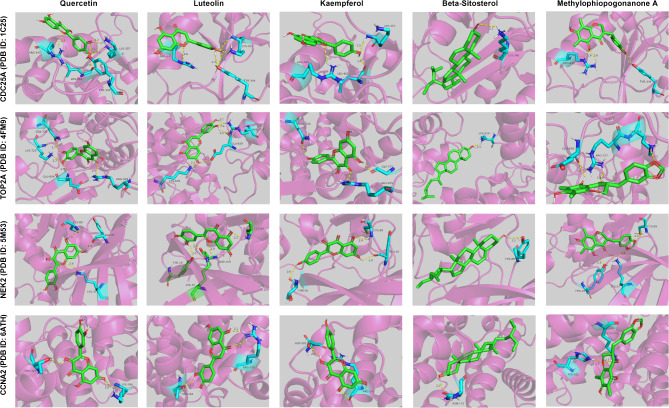
3D molecular docking diagram of key components and hub targets. Green represents key components (ligands), blue represents amino acid residues surrounding the binding bag, and yellow dashed lines represent hydrogen bonds

In order to identify the hub targets of QYD, CytoHubba plugin was used to screen the top 15 key genes in the PPI network through 9 different algorithms (Table 2). As shown in (Fig. 5A), CDC25A, TOP2A, NEK2 and CCNA2 were identified as hub targets for QYD in psoriasis treatment. The detailed information of these four hub genes is shown in Table 3. Genes/proteins exert their biological functions in a complex network. In order to further explore the potential biological effects induced by QYD treatment, based on the GeneMania database, the co-expressed gene interaction network of the 4 hub genes were mapped. Functionally, the hub genes and its co-expressed genes were involved in cell cycle checkpoint, DNA integrity checkpoint, cell cycle G2/M phase transition, and DNA damage checkpoint and signal transduction by p53 class mediator (Fig. 5B). Consistently, GO analysis showed that the hub genes and their co-expressed genes were associated with cell cycle checkpoints and DNA integrity checkpoints (Fig. 5C). KEGG pathway enrichment analysis showed that the hub genes and their co-expressed genes were significantly enriched on p53 signaling pathway and FoxO signaling pathway (Fig. 5D). Next, molecular docking was applied to analyze the possibility of hub targets binding to key components. As shown, the binding energies between quercetin, luteolin, kaempferol, beta-sitosterol and methylophiopogonanone A and the targets were all less than − 6.5 kcal/mol, indicating good binding affinity between key components and hub target genes (Figs. 6 and 7). These results implied that the crucial components of QYD might be the natural inhibitors of the hub targets.

**Table 2:** The top 15 gene rank in CytoHubba

Betweenness	Closeness	Degree	EPC	MCC	DMNC	Radiality	Stress	MNC
REN	CDC25A	CDC25A	CCNA2	CDC25A	NEK2	CDC25A	DHFR	CDC25A
DHFR	CCNA2	CCNA2	CDC25C	CCNA2	MELK	DHFR	MCL1	CCNA2
PTPRC	CDK1	CDK1	CDC25A	CDK1	PLK4	CCNA2	REN	CDK1
MCL1	CCNB1	TOP2A	CCNB1	CCNB1	TTK	CDK1	PTPRC	CCNB1
CDC25A	TOP2A	CCNB1	CDK1	AURKA	AURKA	CCNB1	CDC25A	CDC25C
SCARB1	NEK2	CDC25C	PLK4	TOP2A	TOP2A	NEK2	XDH	TOP2A
XDH	CDC25C	AURKA	TOP2A	PLK4	DBF4	TOP2A	GART	AURKA
G6PD	AURKA	PLK4	MELK	TTK	CCNB1	MCL1	G6PD	PLK4
TOP2A	DHFR	TTK	AURKA	CDC25C	CDC25C	CDC25C	SCARB1	TTK
GART	PLK4	NEK2	TTK	NEK2	MCL1	AURKA	NEK2	NEK2
CSNK1A1	TTK	MELK	NEK2	MELK	CDC25A	G6PD	CCNA2	MELK
PLAT	MELK	DHFR	DBF4	DBF4	CCNA2	GART	CDK1	DBF4
MALT1	MCL1	MCL1	DHFR	MCL1	CDK1	PTPRC	TOP2A	PNP
NEK2	GART	GART	MCL1	PNP	DHFR	PLK4	CCNB1	GART
CCNA2	DBF4	DBF4	GART	GDA	GDA	TTK	PLAT	TYMP

**Table 3:** Hub targets information of Qingying Decoction in the treatment of psoriasis

Gene	CDC25A	TOP2A	NEK2	CCNA2
Uniprot ID	P30304	P11388	P51955	P20248
Betweenness	327.6511597	183.0260812	133.9305594	100.3620331
Closeness	25.04285714	23.57619048	22.57619048	24.20952381
Degree	15	14	11	14
EPC	26.811	26.798	26.712	26.838
MCC	3,669,963	3,669,242	3,628,801	3,669,962
DMNC	0.69819065	0.819558405	0.897868042	0.69819065
Radiality	7.790697674	7.558139535	7.558139535	7.697674419
Stress	984	532	706	592

### QYD can inhibit the excessive proliferation and cell cycle progression of HaCaT keratinocytes

**Fig. 8:**
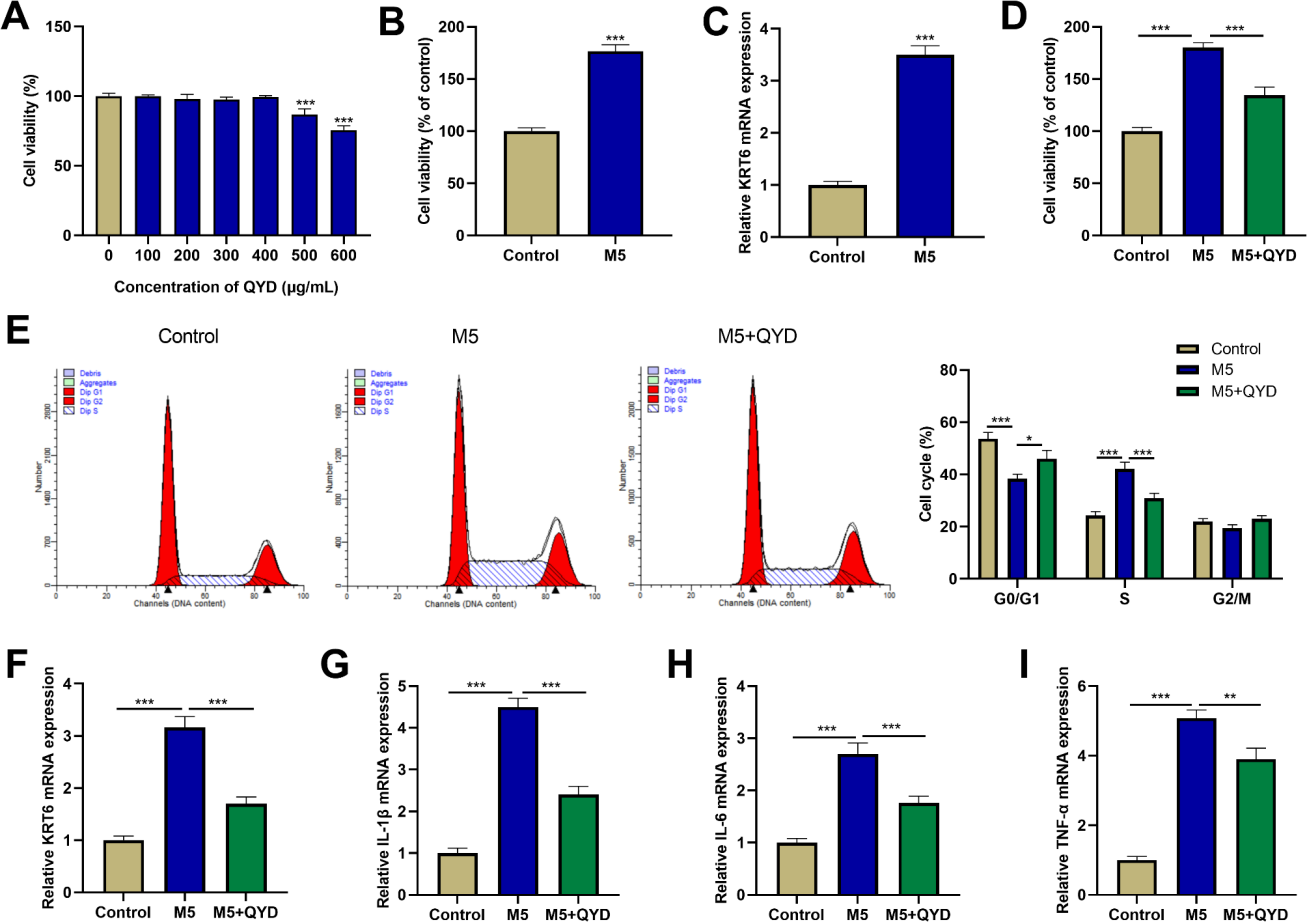
QYD reversed the effects of M5 stimulation on HaCaT keratinocyte viability, cell cycle progression, and inflammatory response. **A**. Effect of QYD on the viability of HaCaT keratinocytes. HaCaT cells were treated with different concentrations of QYD (0, 100, 200, 300, 400, 500, 600 µg/mL) for 24 h. Then the cell viability was measured using CCK-8. **B**&**C**. After M5 (2.5 ng/mL) stimulation of HaCaT cells for 24 h, cell viability was detected by CCK-8 method (**B**), and mRNA expression level of KRT6 was detected by qRT-PCR (**C**). **D**&**E**. After HaCaT cells were stimulated by M5, and treated with QYD, cell viability was detected by CCK-8 (**D**), and the cell cycle was measured by flow cytometry (**E**). **F**-**I**. The mRNA expression levels of KRT6 (**F**), IL-1β (**G**), IL-6 (**H**) and TNF-α (**I**) were detected by qRT-PCR. *P < 0.05, **P < 0.01, ***P < 0.001

Excessive proliferation of keratinocytes and inflammatory response are important factors in the pathogenesis of psoriasis [21]. In order to validate the function and mechanism of QYD in treating psoriasis, CCK-8 method was applied to detect the viability of HaCaT cells treated with QYD. At concentrations below 400 µg/mL, no significant toxicity was observed after 24 h of QYD treatment. At concentrations above 500 µg/mL, QYD significantly reduced HaCaT cell viability (Fig. 8A). Therefore, in this study, the concentration of QYD used was less than 400 µg/mL. M5 (2.5ng /ml) was used to stimulate HaCaT cells for 24 h to establish a psoriatic keratinocyte model in vitro. As shown (Fig. 8B), M5 stimulation significantly promoted the viability of HaCaT cells. KRT6 is known to be a marker of excessive keratinocyte proliferation in psoriasis [22]. qRT-PCR results showed that M5 stimulation significantly increased the mRNA expression level of KRT6 in HaCaT cells (Fig. 8C). Subsequently, the effects of QYD treatment on M5-induced HaCaT cell viability and cell cycle progression were examined. The results showed that QYD treatment significantly reduced M5-induced overproliferation of HaCaT cells (Fig. 8D). Flow cytometry analysis showed that M5 stimulation significantly decreased the number of HaCaT cells in G0/G1 phase and increased the percentage of HaCaT cells in S phase, while QYD treatment attenuated this effect (Fig. 8E). In addition, mRNA expression levels of KRT6 and inflammatory factors (IL-1β, IL-6, and TNF-α) were significantly reduced in HaCaT cells treated with QYD and M5 together compared with M5 alone (Fig. 8F-I).

### QYD inhibits the activation of PI3K/AKT/FoxO pathway in M5-induced HaCaT cells

**Fig. 9:**
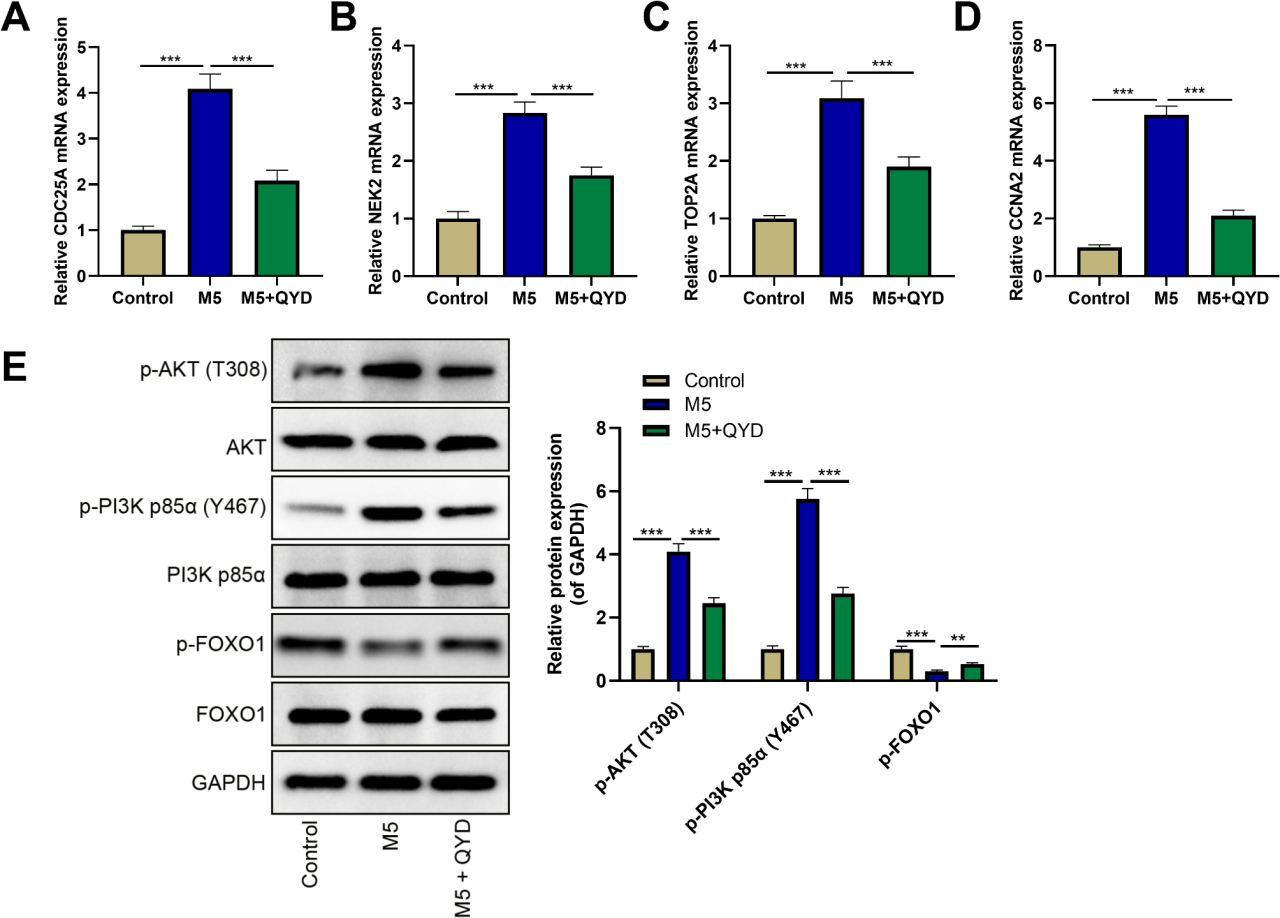
QYD treatment reversed the effects of M5 stimulation on hub targets’ expression and PI3K/AKT/FoxO pathway. **A**–**D**. The mRNA expression levels of CDC25A (**A**), TOP2A (**B**), NEK2 (**C**) and CCNA2 (**D**) were detected by qRT-PCR after HaCaT cells were stimulated by M5 and treated with 400 µg/mL QYD. **E**. The protein expression levels of p-AKT, p-PI3K and p-FOXO1 were detected by Western blot. **P < 0.01, ***P < 0.001

In order to investigate whether the inhibition of keratinocyte proliferation and inflammatory response mediated by QYD was associated with the hub targets, qRT-PCR was used to detect the effect of QYD treatment on the mRNA expression levels of hub genes. The results showed that M5 stimulation significantly up-regulated the mRNA expression levels of CDC25A, TOP2A, NEK2, and CCNA2 in HaCaT cells, while this effect was reversed after QYD treatment (Fig. 9A-D). The PI3K/AKT/FoxO pathway is known to play a crucial role in the pathogenesis of psoriasis [23]. So Western blot was used to detect the effects of QYD treatment on PI3K/AKT/FoxO pathway in HaCaT cells. Compared with the control group, M5 stimulation significantly increased the protein levels of p-AKT and p-PI3K p85α, and decreased the protein levels of p-FOXO1 in HaCaT cells; compared with the M5 group, the levels of p-AKT and p-PI3K p85α protein in the M5 + QYD group were significantly down-regulated, while the levels of p-FOXO1 protein were significantly up-regulated (Fig. 9E). These results suggest that QYD can play a role in the treatment of psoriasis by acting on hub targets including CDC25A, TOP2A, NEK2, CCNA2 and PI3K/AKT/FoxO pathway.

## Discussion

The pathogenesis of psoriasis is complex, which is related to autoimmune system disorders, obesity, and abnormal inflammatory signal transduction [24, 25]. The unknown pathogenesis makes treatment difficult. TCM has unique advantages in the treatment of psoriasis, which can play a therapeutic role through a multi-component, multi-target and multi-pathway [26]. QYD is a classic prescription composed of buffalo horn and eight herbal medicines, which has significant antipyretic effect [27]. It is reported that QYD can effectively alleviate the clinical symptoms of patients with “hematothermal” psoriasis, and it is tried to combine with secukinumab to treat plaque psoriasis [8]. However, its potential mechanism of action in the treatment of psoriasis has not been elucidated. The aim of this study was to explore the potential mechanism of action of QYD in the treatment of psoriasis using network pharmacology, bioinformatics and molecular docking.

Network pharmacology has become a powerful tool to predict the molecular mechanism of drug-psoriasis interaction [28, 29]. However, lack of clinical information may limit the value and application of network pharmacology. WGCNA can classify highly co-expressed genes and connect them into networks [14]. In this study, we used bioinformatics and WGCNA methods to identify 600 genes that are significantly associated with clinical features of psoriasis. Of these, 59 genes have been identified as potential candidates for psoriasis therapy QYD, and these genes are mainly involved in nucleotide metabolism, cell cycle, T cell receptor signaling pathways, purine metabolism, and metabolic pathways. Compared with the healthy control group, the psoriasis group had a higher abundance of B cells naive and dendritic cells activated, which was consistent with previous findings [30]. It is worth mentioning that the network pharmacological analysis suggests that quercetin, luteolin, kaempferol, β-sitosterol and methylophiopogonanone A may be the key components of QYD in the treatment of psoriasis. Quercetin has been reported to improve praziquantimode-induced psoriasis like skin inflammation in mice by regulating the NF-κB pathway [31]. Luteolin is a natural flavonoid with anti-inflammatory activity, which has a good inhibitory effect on keratinocyte proliferation and can effectively relieve psoriasis like skin lesions [32]. Kaempferol is a natural flavonol found in various plants and is considered to be an effective drug in the treatment of psoriasis [33]. Previous studies have confirmed that β-sitosterol can reduce epidermal hyperplasia and immune cell infiltration in mouse model of psoriasis [34]. These findings suggest that QYD can play a role in the treatment of psoriasis through a variety of components. It is important to identify these key bioactive ingredients in order to optimize the formulation of QYD, or to try to use these bioactive ingredients to treat psoriasis and avoid the potential adverse effects of other ingredients in QYD.

We also identified four hub targets of QYD in the treatment of psoriasis, namely CDC25A, TOP2A, NEK2, and CCNA2. CDC25A is a bisspecific phosphatase that promotes cell cycle progression through dephosphorylation of cycle-dependent kinases (CDKs) [35]. It has been proven to be involved in the proliferation and cycle progression of psoriatic keratinocytes [36]. TOP2A protein is a key enzyme in DNA replication and transcription, as well as a proliferative marker of tumor cells [37]. It has been reported that TOP2A is highly expressed in psoriasis, and its high expression is associated with poor prognosis in psoriasis patients [38]. The expression of NEK2 is up-regulated in psoriatic lesions, and its high expression can aggravate the psoriatic dermatitis induced by imiquimote, and promote keratinocyte proliferation and inflammatory response [39]. High expression of CCNA2 is closely related to the high proliferation of keratinocytes [40]. These findings indicate that the hub targets predicted in this study are mainly related to the proliferation and cycle progression of keratinocytes, and preliminarily confirm the possibility of QYD treating psoriasis at the molecular level. Molecular docking technology can reveal the interaction mechanism between the active ingredients of traditional Chinese medicine and the target protein. Through the docking of known small molecule active substances with related target proteins, molecular docking technology can clarify the mechanism of action between TCM effector components and targets at the molecular level, which is of great significance for understanding the pharmacological effects of TCM. Importantly, in the present work, molecular docking analysis showed that the binding energies of the key components of QYD with these hub targets were all less than − 6.5 kcal/mol, indicating that the key components of QYD could effectively bind to the hub target, and implying QYD may play a therapeutic role in psoriasis by regulating these hub targets.

Keratinocytes are long-lived skin cells. Excessive proliferation of keratinocytes and inflammatory response are important factors in the pathogenesis of psoriasis [21]. Keratinocytes induced by a mixture of five cytokines (M5), namely IL-17 A, IL-22, oncostatin M, IL-1α, and TNF-α, have been widely used as in vitro cell models of psoriasis [20, 21, 41]. In the current study, M5 stimulation was found to induce keratinocyte hyperproliferation and inflammatory responses, and to increase mRNA expression levels of KRT6, a marker of keratinocyte hyperproliferation. These findings are consistent with previous studies [20]. Interestingly, QYD treatment mitigated M5-induced hyperproliferation and cycle progression, partially reducing the mRNA expression of KRT6 and inflammatory factors (IL-1β, IL-6, and TNF-α) in keratinocytes. In addition, M5 stimulation significantly increased mRNA expression levels of CDC25A, TOP2A, NEK2, and CCNA2 in keratinocytes, while QYD treatment reversed this effect. KEGG analysis showed that hub gene and its co-expressed genes were significantly enriched on FoxO signaling pathway. FOXO1, a member of the mammalian Forkhead box O (FOXO) transcription factor protein family [42], is a negative regulatory target of the PI3K/AKT pathway. FOXO1 can inhibit cell proliferation by stimulating cell cycle inhibitors such as p27 and p21 [43]. PI3K cascade regulates the proliferation of keratinocytes by stimulating AKT and other substrates and inhibiting FOXO1 [23]. Notably, this study found that QYD treatment significantly inhibited protein levels of p-PI3K and p-AKT and increased protein levels of p-FOXO1 in M5-induced keratinocytes. Taken together, these findings suggest that QYD may mitigate keratinocyte overproliferation and inflammatory response by regulating hub targets’ expression levels and the PI3K/AKT/FOXO1 pathway. Psoriasis is considered a systemic inflammatory condition, which is beyond the skin, and chronic inflammation in the patients lead to an increased incidence of other diseases such as cardiovascular diseases, osteoporosis, depression, etc [44–46]. Some previous studies have emphasized the importance of JAK/STAT3 and NF-κB signaling in the inflammatory response during psoriasis pathogenesis [47, 48]. In the following studies, the regulatory effects of QYD in regulating JAK/STAT3 and NF-κB signaling deserve to be further investigated, in both skin lesion inflammation and systemic inflammation.

There are some limitations in the present work. First of all, the target of QYD in this study relies on SwissTargetPrediction database, but the database mainly contains information about coding genes and lacks information about non-coding RNAs. Recent studies suggest that non-coding RNAs, including long non-coding RNAs and microRNAs (miRNAs), play an important role in the occurrence and development of psoriasis [49, 50]. In the future, the regulatory effects of QYD active ingredients on these non-coding RNAs also need to be further explored. Secondly, in terms of experimental verification, only cell models were used in this study. In the future, animal models will help to further clarify the therapeutic effect and mechanism of QYD on psoriasis.

## Conclusion

QYD plays a promising role in the treatment of psoriasis by acting on CDC25A, TOP2A, NEK2, CCNA2 and other targets, regulating PI3K/AKT/FOXO1 pathway to inhibit excessive proliferation of keratinocytes and inflammatory response, and thus plays an important role in the treatment of psoriasis. In order to better develop the clinical application of QYD, further in vivo experimental validation and clinical trials are needed in the following studies.

## Electronic supplementary material

Below is the link to the electronic supplementary material.

Supplementary Material 1

## Data Availability

The data used to support the findings of this study are available from the corresponding author upon request.

## References

[1] Rendon A, Schäkel K. Psoriasis pathogenesis and treatment. Int J Mol Sci. 2019;20(6):1475. 30909615 10.3390/ijms20061475 PMC6471628

[2] Damiani G, Bragazzi NL, Karimkhani Aksut C, Wu D, Alicandro G, McGonagle D, Guo C, Dellavalle R, Grada A, Wong P, La Vecchia C, Tam LS, Cooper KD, Naghavi M. The global, regional, and National burden of psoriasis: results and insights from the global burden of disease 2019 study. Front Med (Lausanne). 2021;8:743180. 34977058 10.3389/fmed.2021.743180 PMC8716585

[3] Ruggiero A, Fabbrocini G, Cacciapuoti S, Cinelli E, Gallo L, Megna M. Ocular manifestations in psoriasis screening (OcMaPS) questionnaire: A useful tool to reveal misdiagnosed ocular involvement in psoriasis. J Clin Med. 2021;10(5):1031. 33802255 10.3390/jcm10051031 PMC7958956

[4] Carmona-Rocha E, Puig L. The biological basis of disease recurrence in psoriasis. Ital J Dermatol Venerol. 2023;158(4):279–91. 37404193 10.23736/S2784-8671.23.07583-7

[5] Griffiths CEM, Armstrong AW, Gudjonsson JE, Barker JNWN. Psoriasis Lancet. 2021;397(10281):1301–15. 33812489 10.1016/S0140-6736(20)32549-6

[6] Lee HJ, Kim M. Challenges and future trends in the treatment of psoriasis. Int J Mol Sci. 2023;24(17):13313. 37686119 10.3390/ijms241713313 PMC10487560

[7] Yang X, Luo G, Fu L, Huang H, Wang L, Yin L, Zhang X, Wang T, Ma X, Feng T, Ye J. Intervention mechanism of Hunag-Lian Jie-Du Decoction on canonical Wnt/β-Catenin signaling pathway in psoriasis mouse model. Evid Based Complement Alternat Med. 2022;2022:3193572. 35463060 10.1155/2022/3193572 PMC9023143

[8] Tian Z, Cang R, Lei M, et al. Enhanced effect of Qing-Ying Decoction concurrent with Secukinumab in the treatment of plaque psoriasis. Altern Ther Health Med. 2022;28(6):96–102. 35687705

[9] Fu DC, Hua Z, Li YG, Wu HY, Guo XY, Huang JZ. [Treatment of early and mid-term primary biliary cirrhosis by Qingying Huoxue Decoction combined ursodeoxycholic acid: a clinical observation]. Zhongguo Zhong Xi Yi Jie He Za Zhi. 2015;35(3):290–3. Chinese. PMID: 25951632. 25951632

[10] Hopkins AL. Network pharmacology: the next paradigm in drug discovery. Nat Chem Biol. 2008;4(11):682–90. 18936753 10.1038/nchembio.118

[11] Liu Z, Huang H, Yu Y, Li L, Shi X, Wang F. Exploring the mechanism of ellagic acid against gastric cancer based on bioinformatics analysis and network Pharmacology. J Cell Mol Med. 2023;27(23):3878–96. 37794689 10.1111/jcmm.17967 PMC10718161

[12] Zhang B, Horvath S. A general framework for weighted gene co-expression network analysis. Stat Appl Genet Mol Biol. 2005;4:Article17. 16646834 10.2202/1544-6115.1128

[13] Zeng Y, Xiao S, Yang L, Ma K, Shang H, Gao Y, Wang Y, Zhai F, Xiang R. Systematic analysis of the mechanism of Xiaochaihu Decoction in hepatitis B treatment via network Pharmacology and molecular Docking. Comput Biol Med. 2021;138:104894. 34607274 10.1016/j.compbiomed.2021.104894

[14] Wu C, Huang ZH, Meng ZQ, Fan XT, Lu S, Tan YY, You LM, Huang JQ, Stalin A, Ye PZ, Wu ZS, Zhang JY, Liu XK, Zhou W, Zhang XM, Wu JR. A network Pharmacology approach to reveal the Pharmacological targets and biological mechanism of compound Kushen injection for treating pancreatic cancer based on WGCNA and in vitro experiment validation. Chin Med. 2021;16(1):121. 34809653 10.1186/s13020-021-00534-y PMC8607619

[15] Yi M, Nissley DV, McCormick F, Stephens RM. SsGSEA score-based Ras dependency indexes derived from gene expression data reveal potential Ras addiction mechanisms with possible clinical implications. Sci Rep. 2020;10(1):10258. 32581224 10.1038/s41598-020-66986-8 PMC7314760

[16] Chen B, Khodadoust MS, Liu CL, Newman AM, Alizadeh AA. Profiling tumor infiltrating immune cells with CIBERSORT. Methods Mol Biol. 2018;1711:243–59. 29344893 10.1007/978-1-4939-7493-1_12 PMC5895181

[17] Trott O, Olson AJ. AutoDock Vina: improving the speed and accuracy of Docking with a new scoring function, efficient optimization, and multithreading. J Comput Chem. 2010;31(2):455–61. 19499576 10.1002/jcc.21334 PMC3041641

[18] Luo Z, Xia LY, Tang YQ, Huang L, Liu D, Huai WY, Zhang CJ, Wang YQ, Xie YM, Yin QZ, Chen YH, Zhang TE. Action mechanism underlying improvement effect of Fuzi Lizhong Decoction on nonalcoholic fatty liver disease: A study based on network Pharmacology and molecular Docking. Evid Based Complement Alternat Med. 2022;2022:1670014. 35096103 10.1155/2022/1670014 PMC8794673

[19] Wang K, Miao X, Kong F, Huang S, Mo J, Jin C, Zheng Y. Integrating network Pharmacology and experimental verification to explore the mechanism of effect of Zuojin pills in pancreatic cancer treatment. Drug Des Devel Ther. 2021;15:3749–64. 10.2147/DDDT.S323360 PMC8427689 34511884

[20] Gao J, Chen F, Fang H, Mi J, Qi Q, Yang M. Daphnetin inhibits proliferation and inflammatory response in human HaCaT keratinocytes and ameliorates imiquimod-induced psoriasis-like skin lesion in mice. Biol Res. 2020;53(1):48. 33081840 10.1186/s40659-020-00316-0 PMC7576854

[21] Guilloteau K, Paris I, Pedretti N, Boniface K, Juchaux F, Huguier V, Guillet G, Bernard FX, Lecron JC, Morel F. Skin inflammation induced by the synergistic action of IL-17A, IL-22, Oncostatin M, IL-1{alpha}, and TNF-{alpha} recapitulates some features of psoriasis. J Immunol. 2010;184(9):5263–70. 20335534 10.4049/jimmunol.0902464

[22] Yuan LL, Cao CY, Rehmannioside. A inhibits TRAF6/MAPK pathway and improves psoriasis by interfering with the interaction of HaCaT cells with IL-17A. Clin Cosmet Investig Dermatol. 2023;16:2585–96. 10.2147/CCID.S430621 PMC10519428 37752969

[23] Zhang M, Zhang X. The role of PI3K/AKT/FOXO signaling in psoriasis. Arch Dermatol Res. 2019;311(2):83–91. 30483877 10.1007/s00403-018-1879-8

[24] Li J, Yu M, Wang YW, Zhang JA, Ju M, Chen K, Jiang Y, Li M, Chen XS. Prevalence of psoriasis and associated risk factors in China: protocol of a nationwide, population-based, cross-sectional study. BMJ Open. 2019;9(7):e027685. 10.1136/bmjopen-2018-027685 PMC6661637 31345966

[25] Zhou L, Zhang L, Tao D. Investigation on the mechanism of Qubi formula in treating psoriasis based on network Pharmacology. Evid Based Complement Alternat Med. 2020;2020:4683254. 32655662 10.1155/2020/4683254 PMC7327573

[26] Guo S, Zhou JY, Tan C, Shi L, Shi Y, Shi J. Network Pharmacology-Based analysis on the mechanism of action of ephedrae Herba-Cinnamomi ramulus couplet medicines in the treatment for psoriasis. Med Sci Monit. 2021;27:e927421. 33513128 10.12659/MSM.927421 PMC7852043

[27] But PP, Tam YK, Lung LC. Ethnopharmacology of rhinoceros Horn. II: antipyretic effects of prescriptions containing rhinoceros Horn or water Buffalo Horn. J Ethnopharmacol 1991 May-Jun;33(1–2):45–50. 10.1016/0378-8741(91)90159-b 1943172

[28] Wang Z, Zhang HM, Guo YR, Li LL. Molecular mechanisms of Biyu Decoction as treatment for psoriasis: A network Pharmacology and molecular Docking study. World J Clin Cases. 2022;10(21):7224–41. 36158000 10.12998/wjcc.v10.i21.7224 PMC9353920

[29] Guo Y, Gan H, Xu S, Zeng G, Xiao L, Ding Z, Zhu J, Xiong X, Fu Z. Deciphering the mechanism of Xijiao Dihuang Decoction in treating psoriasis by network Pharmacology and experimental validation. Drug Des Devel Ther. 2023;17:2805–19. 10.2147/DDDT.S417954 PMC10504908 37719360

[30] Su W, Wei Y, Huang B, Ji J. Identification of hub genes and immune infiltration in psoriasis by bioinformatics method. Front Genet. 2021;12:606065. 33613635 10.3389/fgene.2021.606065 PMC7886814

[31] Chen H, Lu C, Liu H, Wang M, Zhao H, Yan Y, Han L. Quercetin ameliorates imiquimod-induced psoriasis-like skin inflammation in mice via the NF-κB pathway. Int Immunopharmacol. 2017;48:110–7. 28499194 10.1016/j.intimp.2017.04.022

[32] Zhou W, Hu M, Zang X, Liu Q, Du J, Hu J, Zhang L, Du Z, Xiang Z. Luteolin attenuates imiquimod-induced psoriasis-like skin lesions in BALB/c mice via suppression of inflammation response. Biomed Pharmacother. 2020;131:110696. 32920513 10.1016/j.biopha.2020.110696

[33] Li Y, Cui H, Li S, Li X, Guo H, Nandakumar KS, Li Z. Kaempferol modulates IFN-γ induced JAK-STAT signaling pathway and ameliorates imiquimod-induced psoriasis-like skin lesions. Int Immunopharmacol. 2023;114:109585. 36527884 10.1016/j.intimp.2022.109585

[34] Chang ZY, Chen CW, Tsai MJ, Chen CC, Alshetaili A, Hsiao YT, Fang JY. The Elucidation of structure-activity and structure-permeation relationships for the cutaneous delivery of phytosterols to attenuate psoriasiform inflammation. Int Immunopharmacol. 2023;119:110202. 37075671 10.1016/j.intimp.2023.110202

[35] Wang C, Zeng J, Li LJ, Xue M, He SL. Cdc25A inhibits autophagy-mediated ferroptosis by upregulating ErbB2 through PKM2 dephosphorylation in cervical cancer cells. Cell Death Dis. 2021;12(11):1055. 34743185 10.1038/s41419-021-04342-y PMC8572225

[36] Di Fusco D, Stolfi C, Di Grazia A, Dinallo V, Laudisi F, Marafini I, Colantoni A, Monteleone I, Monteleone G. Albendazole negatively regulates keratinocyte proliferation. Clin Sci (Lond). 2020;134(7):907–20. 32236445 10.1042/CS20191215

[37] Zhang Y, Yang H, Wang L, Zhou H, Zhang G, Xiao Z, Xue X. TOP2A correlates with poor prognosis and affects radioresistance of Medulloblastoma. Front Oncol. 2022;12:918959. 35912241 10.3389/fonc.2022.918959 PMC9337862

[38] Zhu X, Zhang E, Qin L. The high expression of TOP2A and MELK induces the occurrence of psoriasis. Aging. 2024;16. 10.18632/aging.205519 PMC10929818 38382096

[39] Peng Y, Zhang Y, Luo M, Pan Y, Zhou R, Yan YN, Yi T, Luo F, Wang B, Wang L, Ran C, Wang H. NEK2 overexpression aggravates IL-22-induced keratinocyte proliferation and cytokine level increases and IMQ-induced psoriasis-like dermatitis. Biochim Biophys Acta Mol Cell Res. 2023;1870(8):119525. 37348763 10.1016/j.bbamcr.2023.119525

[40] Manczinger M, Kemény L. Novel factors in the pathogenesis of psoriasis and potential drug candidates are found with systems biology approach. PLoS ONE. 2013;8(11):e80751. 24303025 10.1371/journal.pone.0080751 PMC3841158

[41] Chen C, Wu N, Duan Q, Yang H, Wang X, Yang P, Zhang M, Liu J, Liu Z, Shao Y, Zheng Y. C10orf99 contributes to the development of psoriasis by promoting the proliferation of keratinocytes. Sci Rep. 2018;8(1):8590. 29872130 10.1038/s41598-018-26996-z PMC5988722

[42] Aldabbas R, Shaker OG, Ismail MF, Fathy N. miRNA-559 and MTDH as possible diagnostic markers of psoriasis: role of PTEN/AKT/FOXO pathway in disease pathogenesis. Mol Cell Biochem. 2023;478(7):1427–38. 36348199 10.1007/s11010-022-04599-7 PMC10209283

[43] Li J, Yang L, Song L, Xiong H, Wang L, Yan X, Yuan J, Wu J, Li M. Astrocyte elevated gene-1 is a proliferation promoter in breast cancer via suppressing transcriptional factor FOXO1. Oncogene. 2009;28(36):3188–96. 19633686 10.1038/onc.2009.171

[44] Boehncke WH. Systemic inflammation and cardiovascular comorbidity in psoriasis patients: causes and consequences. Front Immunol. 2018;9:579. 29675020 10.3389/fimmu.2018.00579 PMC5895645

[45] Saalbach A, Kunz M. Impact of chronic inflammation in psoriasis on bone metabolism. Front Immunol. 2022;13:925503. 35812457 10.3389/fimmu.2022.925503 PMC9259794

[46] González-Parra S, Daudén E. Psoriasis and depression: the role of inflammation. Actas Dermosifiliogr (Engl Ed). 2019 Jan-Feb;110(1):12–9. 10.1016/j.ad.2018.05.009 30509759

[47] Andrés RM, Hald A, Johansen C, Kragballe K, Iversen L. Studies of Jak/STAT3 expression and signalling in psoriasis identifies STAT3-Ser727 phosphorylation as a modulator of transcriptional activity. Exp Dermatol. 2013;22(5):323–8. 23614738 10.1111/exd.12128

[48] Liu AR, Sarkar N, Cress JD, de Jesus TJ, Vadlakonda A, Centore JT, Griffith AD, Rohr B, McCormick TS, Cooper KD, Ramakrishnan P. NF-κB c-Rel is a critical regulator of TLR7-induced inflammation in psoriasis. EBioMedicine. 2024;110:105452. 39586195 10.1016/j.ebiom.2024.105452 PMC11625363

[49] Freisenhausen JC, Luo L, Kelemen E, Elton J, Skoog V, Pivarcsi A, Sonkoly E. RNA sequencing reveals the long Non-Coding RNA signature in psoriasis keratinocytes and identifies CYDAER as a long Non-Coding RNA regulating epidermal differentiation. Exp Dermatol. 2025;34(2):e70054. 39953783 10.1111/exd.70054 PMC11829188

[50] Di Vincenzo M, Diotallevi F, Piccirillo S, Carnevale G, Offidani A, Campanati A, Orciani M, miRNAs. Mesenchymal stromal cells and major neoplastic and inflammatory skin diseases: A page being written: A systematic review. Int J Mol Sci. 2023;24(10):8502. 37239847 10.3390/ijms24108502 PMC10217999

