# Supplementary material for: Application of bioinformatics analysis and molecular docking to study the mechanism of Qingying decoction in treating psoriasis
Source: Hereditas. 2025 Apr 7;162:52. doi: 10.1186/s41065-025-00421-8 (PMC11974070; doi:10.1186/s41065-025-00421-8)
Supplement: Supplementary file 1 — Supplementary Material 1 [file 41065_2025_421_MOESM1_ESM.docx]

**Supplementary Table 1. Details of active compounds in Qingying Decoction.**

| **NO.** | **Herb** | **Component** | **PubChem id** | **Target** | **Structure** |
| --- | --- | --- | --- | --- | --- |
| 1 | Salvia miltiorrhiza, Radix Scrophulariae | Sugiol | 94162 | 49 | 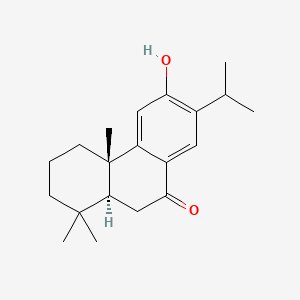 |
| 2 | Lonicerae japonicae flos, Fructus Forsythiae, Radix Scrophulariae, Radix Rehmanniae | Beta-Sitosterol | 222284 | 44 | 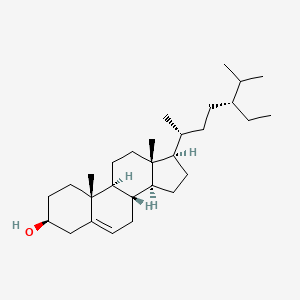 |
| 3 | Radix Scrophulariae | Scropolioside D | 101239919 | 21 | 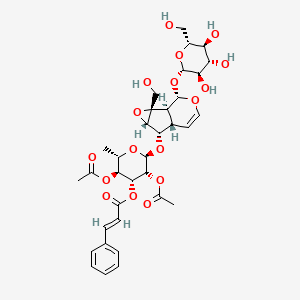 |
| 4 | Buffalo Horn | 4-Guanidino-1-butanol | 5317848 | 5 | 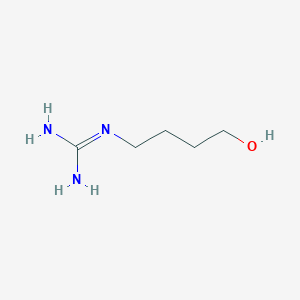 |
| 5 | Buffalo Horn | D-alanine | 71080 | 1 | 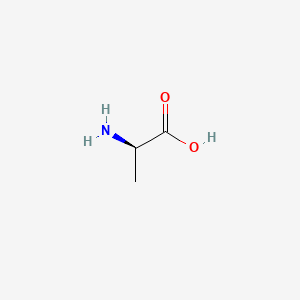 |
| 6 | Buffalo Horn | Arginine | 6322 | 2 | 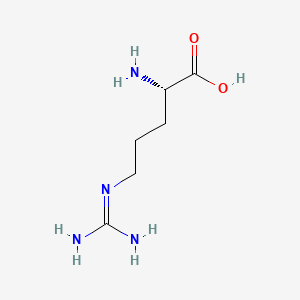 |
| 7 | Buffalo Horn | D-aspartic acid | 83887 | 1 | 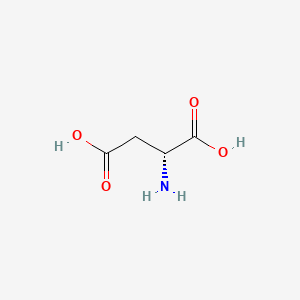 |
| 8 | Buffalo Horn | Cholesterol | 5997 | 56 | 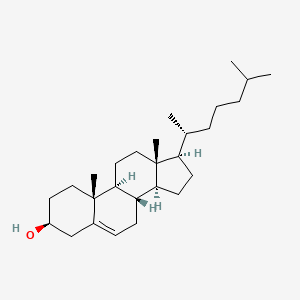 |
| 9 | Radix Rehmanniae | 3-indolecarboxylic acid | 69867 | 9 | 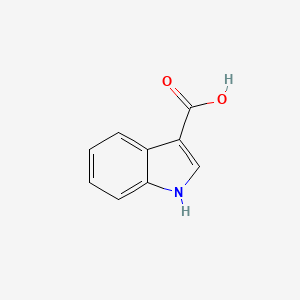 |
| 10 | Radix Rehmanniae | 6-(Hydroxymethyl)pyridin-3-ol | 419490 | 1 | 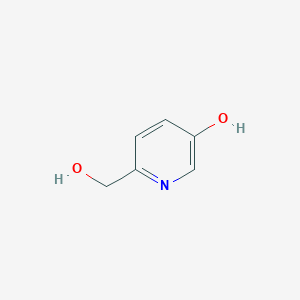 |
| 11 | Radix Rehmanniae | 5-Hydroxy-2-methylpyridine | 14275 | 3 | 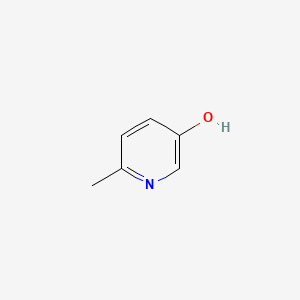 |
| 12 | Radix Rehmanniae | 7-Hydroxyisoquinoline | 459767 | 26 | 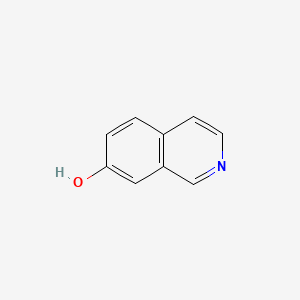 |
| 13 | Radix Rehmanniae | Verbascoside | 5281800 | 10 | 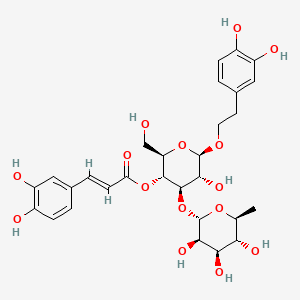 |
| 14 | Radix Rehmanniae | Adenine | 190 | 5 | 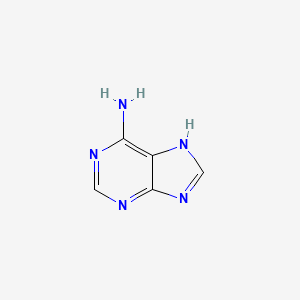 |
| 15 | Radix Rehmanniae, Ophiopogon japonicus | Adenosine | 60961 | 69 | 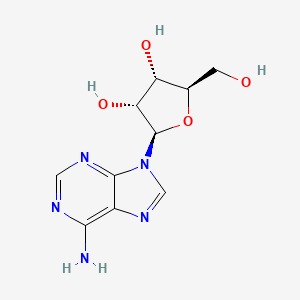 |
| 16 | Radix Rehmanniae | Campesterol | 134766514 | 18 | 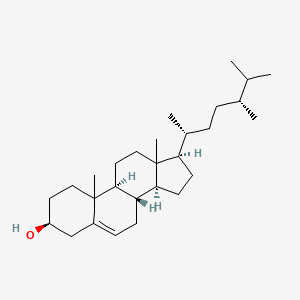 |
| 17 | Radix Rehmanniae | Catalpol | 138107792 | 20 | 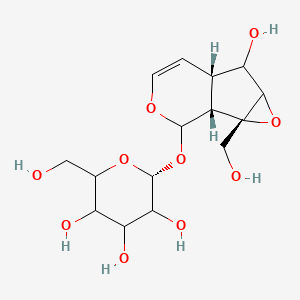 |
| 18 | Radix Rehmanniae | Coniferin | 5280372 | 28 | 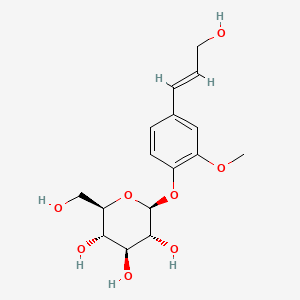 |
| 19 | Radix Rehmanniae | Daucosterol | 5742590 | 31 | 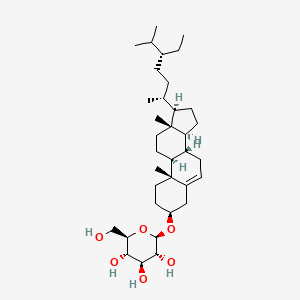 |
| 20 | Radix Rehmanniae | Diincarvilone A | 60155322 | 112 | 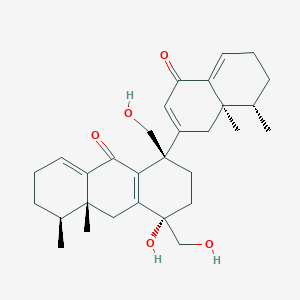 |
| 21 | Radix Rehmanniae | Echinacoside | 5281771 | 6 | 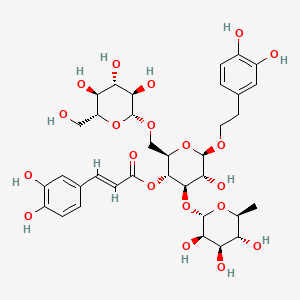 |
| 22 | Radix Rehmanniae | Methyl ferulate | 5357283 | 104 | 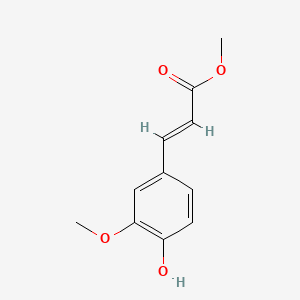 |
| 23 | Radix Rehmanniae | Glutinoside | 21637654 | 13 | 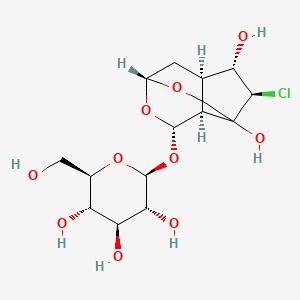 |
| 24 | Radix Rehmanniae, Ophiopogon japonicus | Guanosine | 135398635 | 48 | 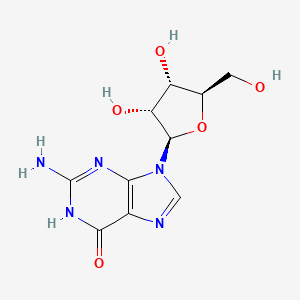 |
| 25 | Radix Rehmanniae | Isoacteoside | 6476333 | 18 | 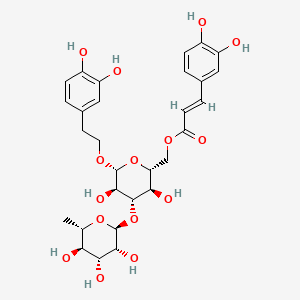 |
| 26 | Radix Rehmanniae | Leonoside F | 57325811 | 6 | 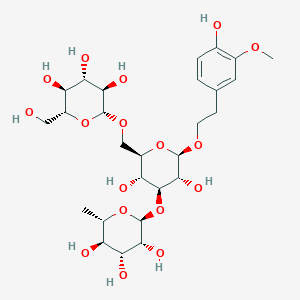 |
| 27 | Radix Rehmanniae | Martynoside | 5319292 | 6 | 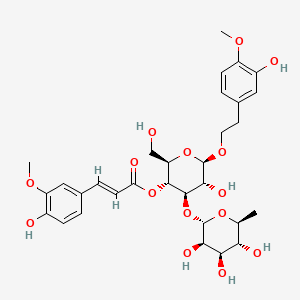 |
| 28 | Radix Rehmanniae | 5-Methoxypyrrolidin-2-one | 181561 | 1 | 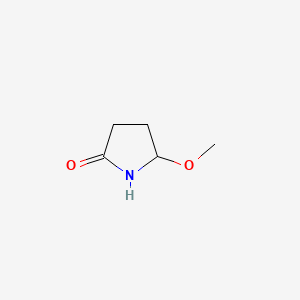 |
| 29 | Radix Rehmanniae | Rehmaglutin A | 5320903 | 40 | 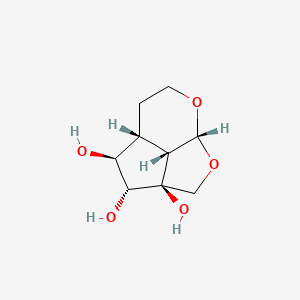 |
| 30 | Radix Rehmanniae | Rehmaglutin B | 14413769 | 17 | 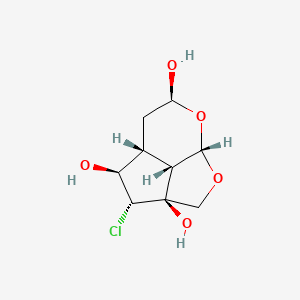 |
| 31 | Radix Rehmanniae | Rehmaglutin C | 21637649 | 37 | 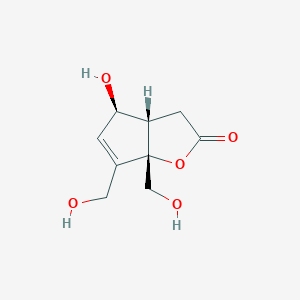 |
| 32 | Radix Rehmanniae | Rehmaglutin D | 5320906 | 14 | 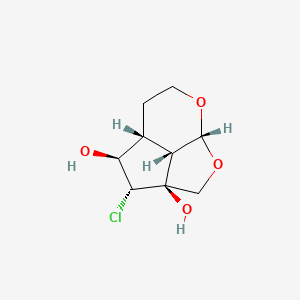 |
| 33 | Radix Rehmanniae | Rehmaionoside A | 10023290 | 75 | 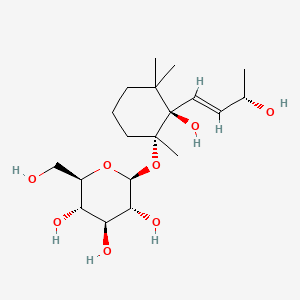 |
| 34 | Radix Rehmanniae | Rehmannio-side D | 138114722 | 4 | 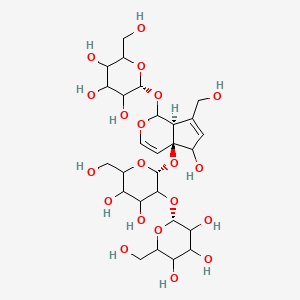 |
| 35 | Radix Rehmanniae | Rehmapicrogenin | 15693864 | 78 | 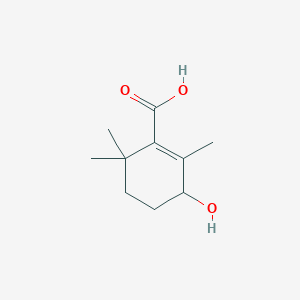 |
| 36 | Radix Rehmanniae | Salidroside | 159278 | 17 | 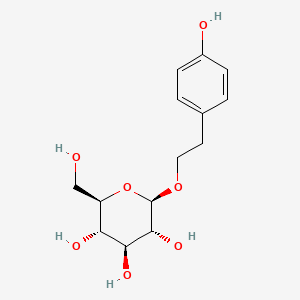 |
| 37 | Radix Rehmanniae, Ophiopogon japonicus | Uridine | 6029 | 26 | 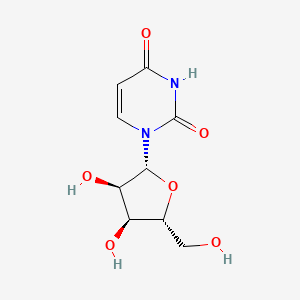 |
| 38 | Ophiopogon japonicus | 5,7-dihydroxy-6,8-dime thyl-3-(4'-hydroxy-3'-methoxybenzyl)chroman-4-one | 5316771 | 97 | 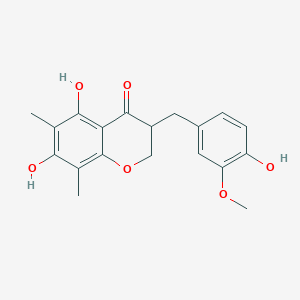 |
| 39 | Ophiopogon japonicus | Jasmolone | 5374699 | 108 | 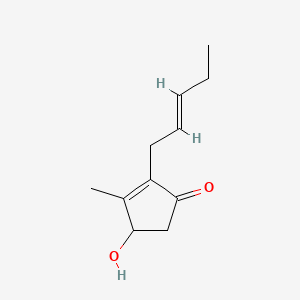 |
| 40 | Ophiopogon japonicus | Methylophiopogonanone A | 53466984 | 111 | 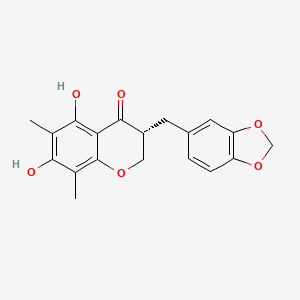 |
| 41 | Ophiopogon japonicus | Methylophiopogonanone B | 46886732 | 103 | 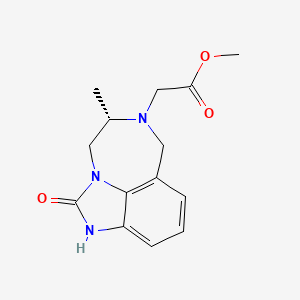 |
| 42 | Ophiopogon japonicus | β-patchoulene | 101731 | 11 | 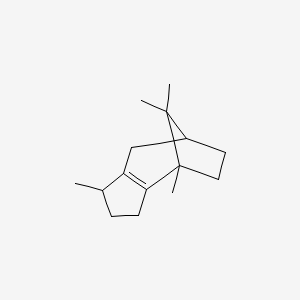 |
| 43 | Ophiopogon japonicus | Diosgenin | 137704703 | 70 | 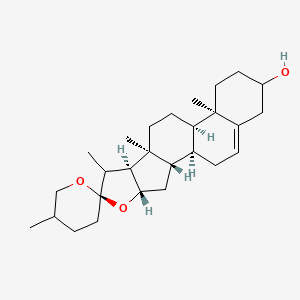 |
| 44 | Ophiopogon japonicus | Methylophiopogonone A | 10065830 | 103 | 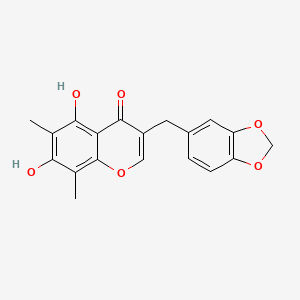 |
| 45 | Ophiopogon japonicus | Methylophiopogonone B | 23259413 | 37 | 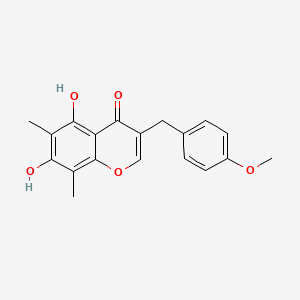 |
| 46 | Ophiopogon japonicus | Oleanolic Acid | 49867939 | 89 | 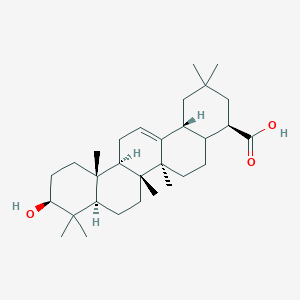 |
| 47 | Ophiopogon japonicus | Ophiopogonanone A | 9996586 | 104 | 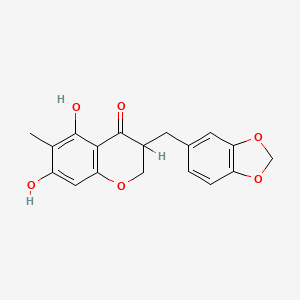 |
| 48 | Ophiopogon japonicus | Ophiopogonanone B | 76036450 | 110 | 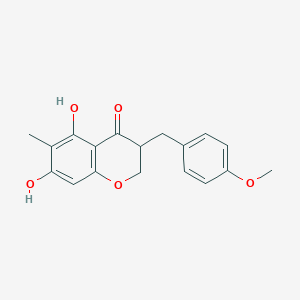 |
| 49 | Ophiopogon japonicus | Ophiopogonanone C | 10871974 | 37 | 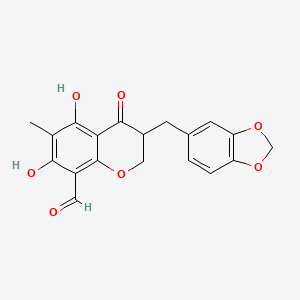 |
| 50 | Ophiopogon japonicus | Ophiopogonanone D | 11003181 | 51 | 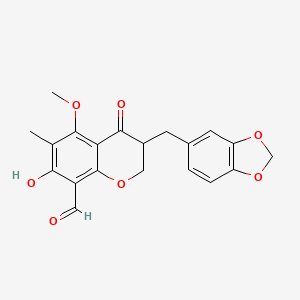 |
| 51 | Ophiopogon japonicus | Ophiopogonanone E | 5316797 | 63 | 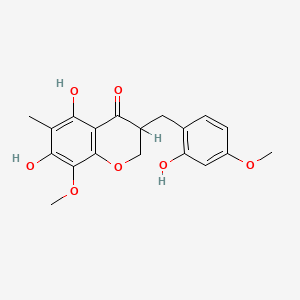 |
| 52 | Ophiopogon japonicus | Ophiopogonanone F | 5318201 | 69 | 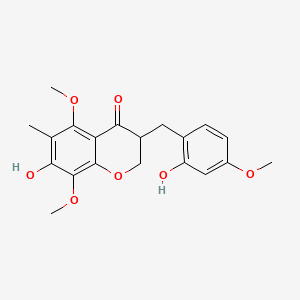 |
| 53 | Ophiopogon japonicus | Ophiopogonin A | 145706175 | 31 | 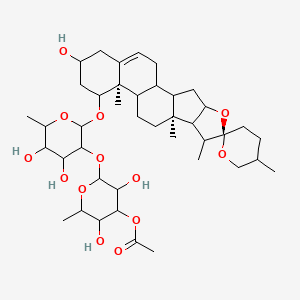 |
| 54 | Ophiopogon japonicus | Ophiopogonin B | 71307569 | 43 | 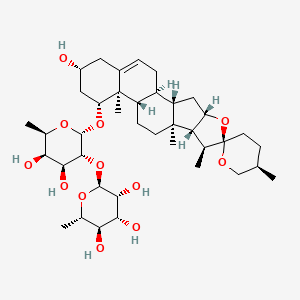 |
| 55 | Ophiopogon japonicus | Ophiopogonin C' | 4483248 | 18 | 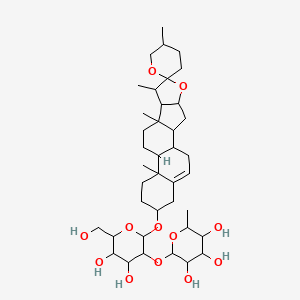 |
| 56 | Ophiopogon japonicus | Ophiopogonin D | 46173859 | 21 | 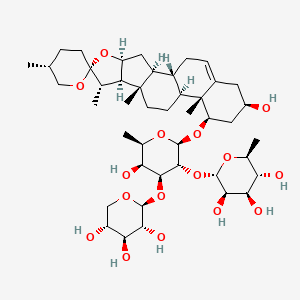 |
| 57 | Ophiopogon japonicus | Ophiopogonin D' | 10033524 | 14 | 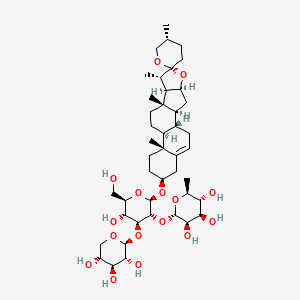 |
| 58 | Ophiopogon japonicus | Ophiopogonone A | 10087732 | 22 | 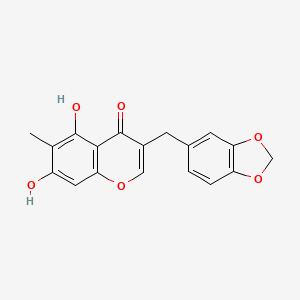 |
| 59 | Ophiopogon japonicus | Ophiopogonone B | 14826840 | 33 | 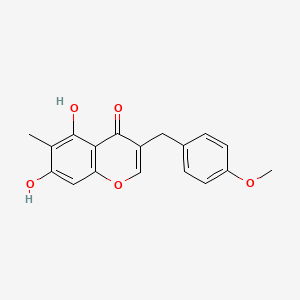 |
| 60 | Ophiopogon japonicus | Ophiopogonone C | 11142766 | 4 | 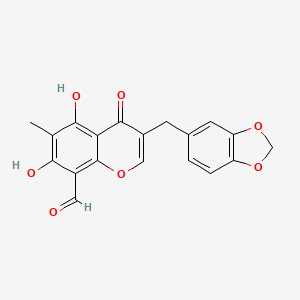 |
| 61 | Ophiopogon japonicus | Ophiopogonoside A | 134715166 | 11 | 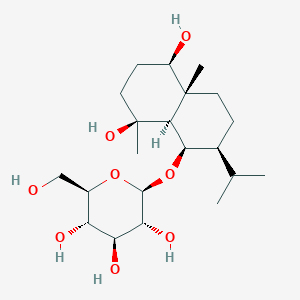 |
| 62 | Ophiopogon japonicus | Orchinol | 181686 | 114 | 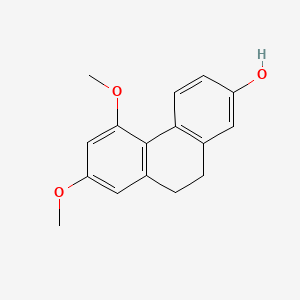 |
| 63 | Ophiopogon japonicus | Ruscogenin | 441893 | 99 | 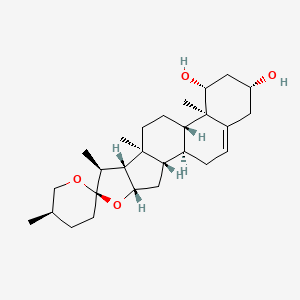 |
| 64 | Lonicerae japonicae flos, Ophiopogon japonicus | Stigmasterol | 5280794 | 41 | 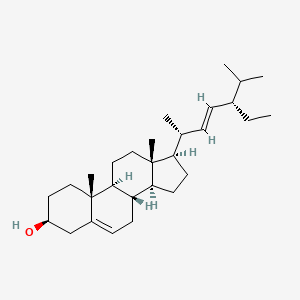 |
| 65 | Fructus Forsythiae | Wogonin | 5281703 | 103 | 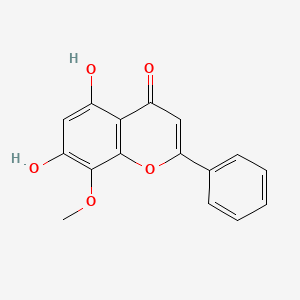 |
| 66 | Fructus Forsythiae | Arctigenin methyl ether | 384877 | 103 | 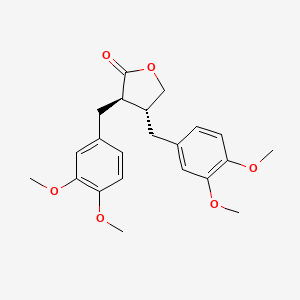 |
| 67 | Fructus Forsythiae | Phillyrin | 101712 | 7 | 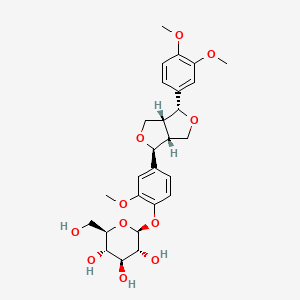 |
| 68 | Fructus Forsythiae | ACon1_001697 | 21722915 | 22 | 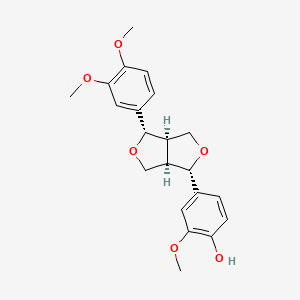 |
| 69 | Fructus Forsythiae | Betulinic Acid | 64971 | 53 | 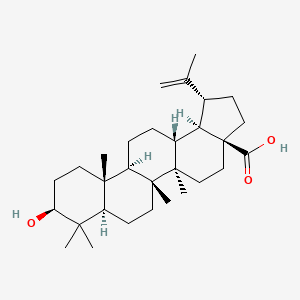 |
| 70 | Fructus Forsythiae | Phillygenol | 4166098 | 22 | 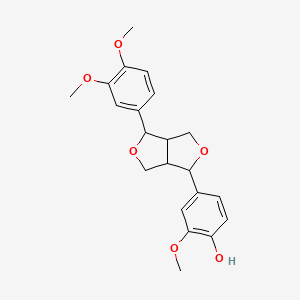 |
| 71 | Fructus Forsythiae | Hyperforin | 441298 | 16 | 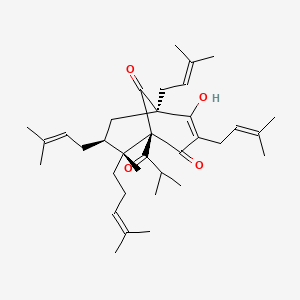 |
| 72 | Fructus Forsythiae | Adhyperforin | 44427225 | 2 | 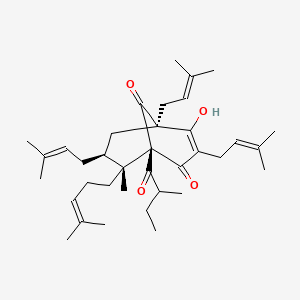 |
| 73 | Fructus Forsythiae | Onjixanthone I | 5320290 | 105 | 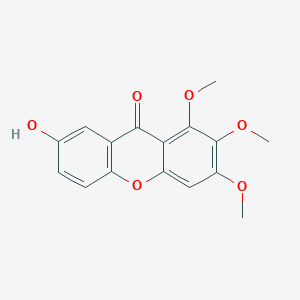 |
| 74 | Lonicerae japonicae flos, Fructus Forsythiae | Kaempferol | 5280863 | 103 | 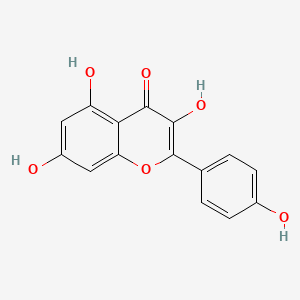 |
| 75 | Fructus Forsythiae | Arctiin | 100528 | 33 | 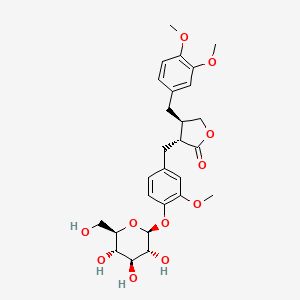 |
| 76 | Salvia miltiorrhiza, Lonicerae japonicae flos, Fructus Forsythiae | Luteolin | 5280445 | 103 | 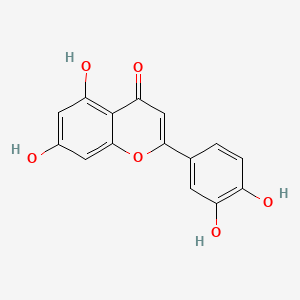 |
| 77 | Fructus Forsythiae | Bicuculline | 10237 | 104 | 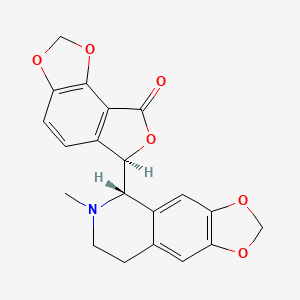 |
| 78 | Rhizoma Coptidis, Lonicerae japonicae flos, Fructus Forsythiae | Quercetin | 5280343 | 103 | 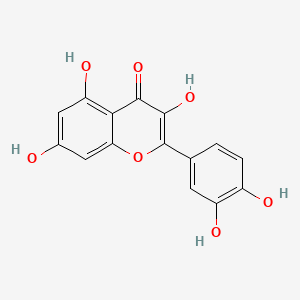 |
| 79 | Lonicerae japonicae flos | Mandenol | 5282184 | 108 | 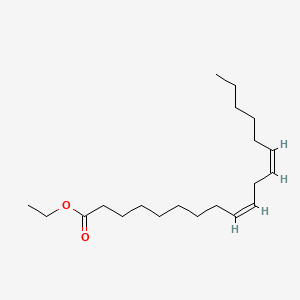 |
| 80 | Lonicerae japonicae flos | Ethyl linolenate | 6371716 | 92 | 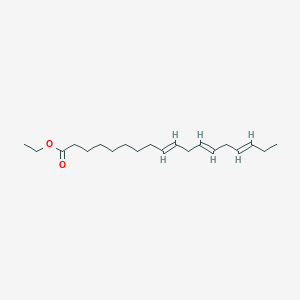 |
| 81 | Lonicerae japonicae flos | Beta-Carotene | 5280489 | 1 | 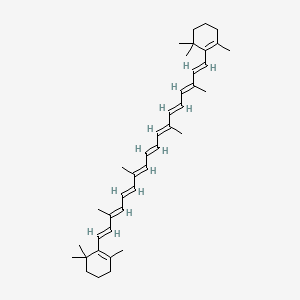 |
| 82 | Lonicerae japonicae flos | Eriodyctiol (flavanone) | 373261 | 80 | 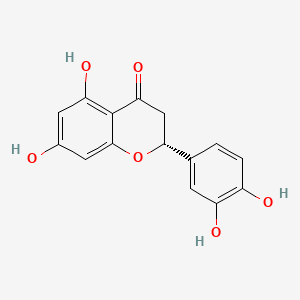 |
| 83 | Lonicerae japonicae flos | ZINC03978781 | 11870462 | 41 | 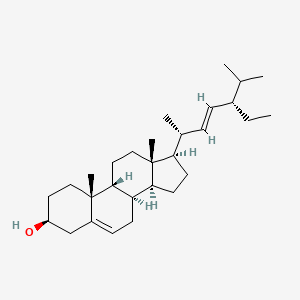 |
| 84 | Lonicerae japonicae flos | Chryseriol | 5280666 | 103 | 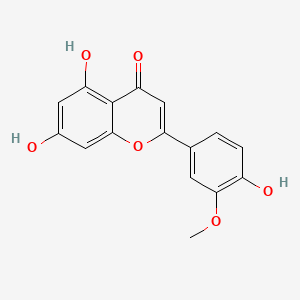 |
| 85 | Lonicerae japonicae flos | Kryptoxanthin | 5281235 | 17 | 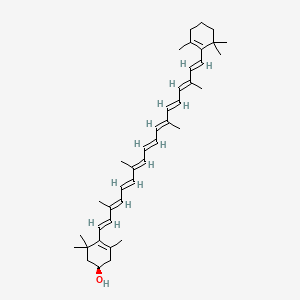 |
| 86 | Lonicerae japonicae flos | all-trans-Rhodoxanthin | 5380108 | 15 | 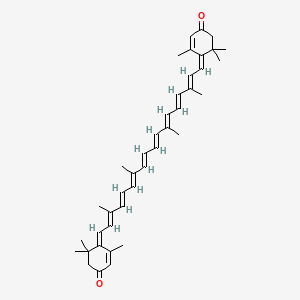 |
| 87 | Lonicerae japonicae flos | Corymbosin | 10970376 | 103 | 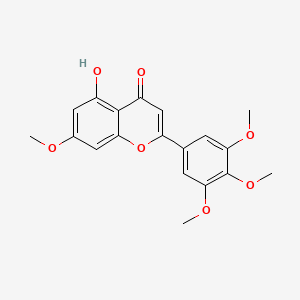 |
| 88 | Lonicerae japonicae flos | XYLOSTOSIDINE | 14466553 | 40 | 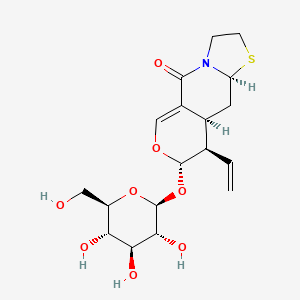 |
| 89 | Rhizoma Coptidis | Berberine | 2353 | 105 | 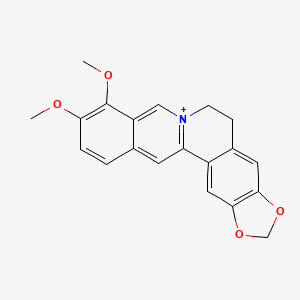 |
| 90 | Rhizoma Coptidis | Obacunone | 119041 | 106 | 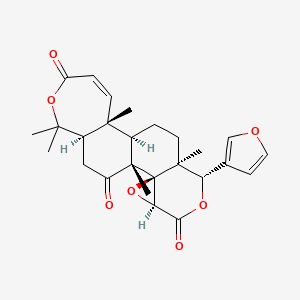 |
| 91 | Rhizoma Coptidis | Berberrubine | 72704 | 23 | 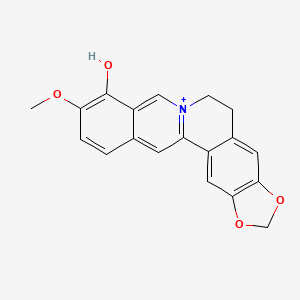 |
| 92 | Rhizoma Coptidis | Epiberberine | 160876 | 102 | 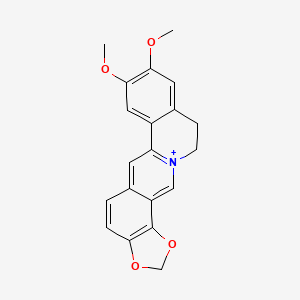 |
| 93 | Rhizoma Coptidis | (R)-Canadine | 443422 | 106 | 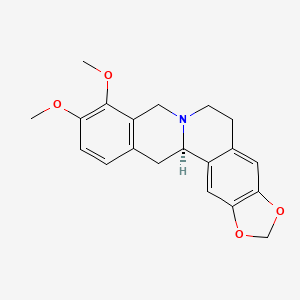 |
| 94 | Rhizoma Coptidis | Berlambine | 11066 | 107 | 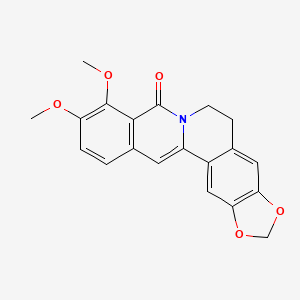 |
| 95 | Rhizoma Coptidis | Magnograndiolide | 5319198 | 50 | 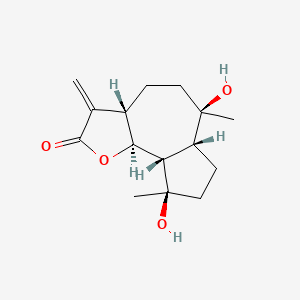 |
| 96 | Rhizoma Coptidis | Palmidin A | 5320384 | 34 | 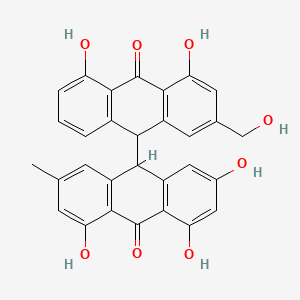 |
| 97 | Rhizoma Coptidis | Palmatine | 19009 | 102 | 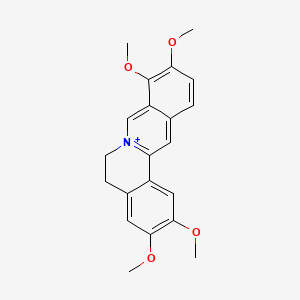 |
| 98 | Rhizoma Coptidis | Coptisine | 72322 | 24 | 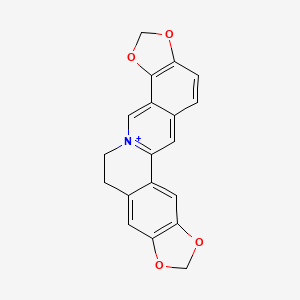 |
| 99 | Radix Rehmanniae, Rhizoma Coptidis, Ophiopogon japonicus | Moupinamide | 5280537 | 104 | 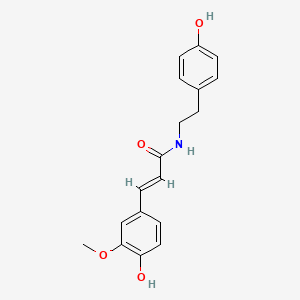 |
| 100 | Salvia miltiorrhiza | Poriferasterol | 5281330 | 41 | 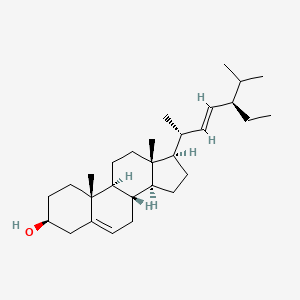 |
| 101 | Salvia miltiorrhiza | Clionasterol | 457801 | 44 |  |
| 102 | Salvia miltiorrhiza | Isoimperatorin | 68081 | 49 |  |
| 103 | Salvia miltiorrhiza | Dehydrotanshinone II A | 128994 | 58 |  |
| 104 | Salvia miltiorrhiza | Baicalin | 64982 | 15 |  |
| 105 | Salvia miltiorrhiza | Digallic acid | 341 | 18 |  |
| 106 | Salvia miltiorrhiza | α-amyrin | 12358389 | 59 |  |
| 107 | Salvia miltiorrhiza | Arucadiol | 11011966 | 90 |  |
| 108 | Salvia miltiorrhiza | 2-Isopropyl-8-methylphenanthrene-3,4-dione | 135872 | 77 |  |
| 109 | Salvia miltiorrhiza | (E)-3-[2-(3,4-dihydroxyphenyl)-7-hydroxy-benzofuran-4-yl]acrylic acid | 10403222 | 20 |  |
| 110 | Salvia miltiorrhiza | 4-methylenemiltirone | 14609851 | 34 |  |
| 111 | Salvia miltiorrhiza | 2-(4-hydroxy-3-methoxyphenyl)-5-(3-hydroxypropyl)-7-methoxy-3-benzofurancarboxaldehyde | 6709746 | 100 |  |
| 112 | Salvia miltiorrhiza | Formyltanshinone | 14609847 | 32 |  |
| 113 | Salvia miltiorrhiza | Methylenetanshinquinone | 105118 | 59 |  |
| 114 | Salvia miltiorrhiza | Przewalskin B | 16102114 | 78 |  |
| 115 | Salvia miltiorrhiza | Przewaquinone C | 126071 | 103 |  |
| 116 | Salvia miltiorrhiza | Sclareol | 163263 | 110 |  |
| 117 | Salvia miltiorrhiza | Tanshinaldehyde | 124268 | 111 |  |
| 118 | Salvia miltiorrhiza | Danshenol B | 3083515 | 108 |  |
| 119 | Salvia miltiorrhiza | Danshenol A | 3083514 | 109 |  |
| 120 | Salvia miltiorrhiza | Salvilenone | 389885 | 24 |  |
| 121 | Salvia miltiorrhiza | Cryptotanshinone | 160254 | 68 |  |
| 122 | Salvia miltiorrhiza | Deoxyneocryptotanshinone | 15690458 | 32 |  |
| 123 | Salvia miltiorrhiza | 3,9-Dimethyl-2,3-dihydrophenanthro[1,2-b]furan-4,5-dione | 40785034 | 65 |  |
| 124 | Salvia miltiorrhiza | Ferruginol | 442027 | 47 |  |
| 125 | Salvia miltiorrhiza | Isotanshinone IIA | 626354 | 14 |  |
| 126 | Salvia miltiorrhiza | Microstegiol | 403772 | 42 |  |
| 127 | Salvia miltiorrhiza | 1-Hydroxy-8,8-dimethyl-2-propan-2-yl-6,7-dihydrophenanthrene-3,4,5-trione | 5319835 | 23 |  |
| 128 | Salvia miltiorrhiza | (1R)-5-hydroxy-1,6,6-trimethyl-2,7,8,9-tetrahydro-1H-naphtho[1,2-g][1]benzofuran-10,11-dione | 5319836 | 42 |  |
| 129 | Salvia miltiorrhiza | Miltipolone | 10086184 | 13 |  |
| 130 | Salvia miltiorrhiza | Miltirone | 160142 | 26 |  |
| 131 | Salvia miltiorrhiza | (4Z)-5-morpholin-4-yl-2-(4-nitrophenyl)-4-(pyridin-3-ylmethylidene)pyrazol-3-one | 5340066 | 38 |  |
| 132 | Salvia miltiorrhiza | Neocryptotanshinone | 44425165 | 39 |  |
| 133 | Salvia miltiorrhiza | 1-methyl-8,9-dihydro-7H-naphtho[5,6-g]benzofuran-6,10,11-trione | 10062187 | 54 |  |
| 134 | Salvia miltiorrhiza | (2R)-3-(3,4-dihydroxyphenyl)-2-[(Z)-3-(3,4-dihydroxyphenyl)acryloyl]oxy-propionic acid | 9841799 | 56 |  |
| 135 | Salvia miltiorrhiza | (Z)-3-[2-[(E)-2-(3,4-dihydroxyphenyl)vinyl]-3,4-dihydroxy-phenyl]acrylic acid | 11602192 | 29 |  |
| 136 | Salvia miltiorrhiza | Salviolone | 10355691 | 22 |  |
| 137 | Salvia miltiorrhiza | Tanshinone IIA | 164676 | 46 |  |
| 138 | Salvia miltiorrhiza | (6S)-6-(hydroxymethyl)-1,6-dimethyl-8,9-dihydro-7H-naphtho[8,7-g]benzofuran-10,11-dione | 9926694 | 105 |  |
| 139 | Salvia miltiorrhiza | Tanshinone VI | 149138 | 44 |  |
